# A Community in Life and Death: The Late Neolithic Megalithic Tomb at Alto de Reinoso (Burgos, Spain)

**DOI:** 10.1371/journal.pone.0146176

**Published:** 2016-01-20

**Authors:** Kurt W. Alt, Stephanie Zesch, Rafael Garrido-Pena, Corina Knipper, Anna Szécsényi-Nagy, Christina Roth, Cristina Tejedor-Rodríguez, Petra Held, Íñigo García-Martínez-de-Lagrán, Denise Navitainuck, Héctor Arcusa Magallón, Manuel A. Rojo-Guerra

**Affiliations:** 1 Danube Private University, Krems, Austria; 2 Institute for Prehistory and Archaeological Science and Hightech Research Center, Basel University, Basel, Switzerland; 3 State Office for Heritage Management and Archaeology Saxony-Anhalt and State Museum of Prehistory, Halle, Germany; 4 German Mummy Project, Reiss-Engelhorn-Museen, Mannheim, Germany; 5 Department of Prehistory and Archaeology, Faculty of Philosophy and Letters, Autonomous University of Madrid, Madrid, Spain; 6 Curt Engelhorn Centre Archaeometry gGmbH, Mannheim, Germany; 7 Laboratory of Archaeogenetics, Institute of Archaeology, Research Centre for the Humanities, Hungarian Academy of Sciences, Budapest, Hungary; 8 Institute of Anthropology, Mainz University, Mainz, Germany; 9 Arcadia-General Foundation of Valladolid University, Valladolid, Spain; 10 Department of Applied and Analytical Paleontology, Mainz University, Mainz, Germany; 11 Department of Prehistory, University of the Basque Government, Vitoria, Spain; 12 Laboratoire TRACES UMR5608, Université de Toulouse, Toulouse, France; 13 Private Technical Archaeologist, Zaragoza, Spain; 14 Department of Prehistory and Archaeology, Valladolid University, Valladolid, Spain; University of Otago, NEW ZEALAND

## Abstract

The analysis of the human remains from the megalithic tomb at Alto de Reinoso represents the widest integrative study of a Neolithic collective burial in Spain. Combining archaeology, osteology, molecular genetics and stable isotope analysis (^87^Sr/^86^Sr, δ^15^N, δ^13^C) it provides a wealth of information on the minimum number of individuals, age, sex, body height, pathologies, mitochondrial DNA profiles, kinship relations, mobility, and diet. The grave was in use for approximately one hundred years around 3700 cal BC, thus dating from the Late Neolithic of the Iberian chronology. At the bottom of the collective tomb, six complete and six partial skeletons lay in anatomically correct positions. Above them, further bodies represented a subsequent and different use of the tomb, with almost all of the skeletons exhibiting signs of manipulation such as missing skeletal parts, especially skulls. The megalithic monument comprised at least 47 individuals, including males, females, and subadults, although children aged 0–6 years were underrepresented. The skeletal remains exhibited a moderate number of pathologies, such as degenerative joint diseases, healed fractures, cranial trauma, and a low intensity of caries. The mitochondrial DNA profiles revealed a pattern pointing to a closely related local community with matrilineal kinship patterns. In some cases adjacent individuals in the bottom layer showed familial relationships. According to their strontium isotope ratios, only a few individuals were likely to have spent their early childhood in a different geological environment, whilst the majority of individuals grew up locally. Carbon and nitrogen isotope analysis, which was undertaken to reconstruct the dietary habits, indicated that this was a homogeneous group with egalitarian access to food. Cereals and small ruminants were the principal sources of nutrition. These data fit in well with a lifestyle typical of sedentary farming populations in the Spanish Meseta during this period of the Neolithic.

## Introduction

Once the Neolithic had been established in the Middle East the new way of life spread from the 7^th^ millennium BC onwards via different routes into Europe. The earliest evidence of farming and animal husbandry on the Iberian Peninsula dates from as early as the 6^th^ millennium BC and is found in the eastern Mediterranean coastal regions, southern Spain and to a lesser extent in the interior [1–5]. Towards the end of the 6^th^ millennium farming had already spread throughout almost the entire peninsula [5]. The Neolithic on the Iberian Peninsula can be divided into an early (5700−4500 cal BC), a middle (4500−4000 cal BC) and a late (4000−3000 cal BC) phase [6]. The earliest evidence of pulses and cereals found so far was unearthed in the interior of Iberia at La Lámpara, La Revilla del Campo, and La Vaquera [2,4]. This early phase of agriculture already comprised a wide range of farming produce: wheat, barley, pulses (peas, lentils, beans) as well as vetch, flax, and poppy. The considerable variety of agricultural crops that have different requirements with regard to their cultivation, processing and use shows that these early farmers already had extensive knowledge of farming [2].

The Neolithic also introduced new funerary rites into Western Europe. The first so-called megalithic tombs were built in Spain, Portugal and France using large blocks and slabs of stone. Soon thereafter similar megalithic tombs appeared along the European Atlantic coast, in Britain and Ireland, Scandinavia and the European interior [7]. In the interior of the Iberian Peninsula these tombs began to appear at the end of the 5^th^ millennium BC [8] and spread throughout the entire region over the course of the 4^th^ millennium BC [5]. The funerary monuments were extremely varied in their construction [5]. A characteristic feature of megalithic tombs is that these were frequented and used permanently and over an extended period of time as collective burial places for the members of a community whilst also serving as venues for ritual acts. Complex patterns of “treating” and “reburying” the skeletal remains have been identified in some megalithic tombs [9].

Skeletal remains are valuable biogenic archives for the study of the human past [10–11]. In this investigation we chose an integrative approach that combined archaeological and anthropological methods to explore the lifestyles and living conditions of the burial community. The osteological findings provided information about the minimum number of individuals (MNI), age, sex, body height, stress markers, diseases, traces of violence, and activity patterns [12–14]. Molecular genetic (mtDNA) data made biological relationships visible and revealed the genetic profile and relations of the individuals buried at Alto de Reinoso with other Iberian and European Neolithic populations [15–16]. Strontium isotope ratios of enamel and bone are geological tracers and were analysed as an indicator of mobility and change of residence [17]. The ^87^Sr/^86^Sr ratios of the trace element strontium in bedrock vary according to the kinds and ages of geological units. The biologically available strontium is transferred through the food chain, stored in enamel during tooth development, and remains afterwards unchanged. Under the precondition of consumption of locally produced food, strontium isotope ratios in enamel that differ from the local baselines can identify non-local individuals in burial communities. The light stable isotope ratios of carbon (δ^13^C) and nitrogen (δ^15^N) reflect dietary habits [18]. Isotope fractionation allows for estimations of the proportions of C_3_ and C_4_ plants in the human diet as well as the contribution of meat, dairy products, or fish. Other studies with a similarly comprehensive approach to reconstructing the past have been published in recent years for different regions and epochs [19–22]. However, similarly extensive presentations of the research results in collective graves are still generally quite rare and completely lacking for Spain [24–25].

The analysis of the skeletal remains from the megalithic tomb at Alto de Reinoso is therefore the most wide-ranging study of the Neolithic in Spain. First of all, it aims to characterise the population group who buried their dead in the megalithic tomb. Did it comprise several generations of a village-like community with a restricted territory and indications of kinship relations, or do the genetic, isotopic, and osteological characteristics point to the presence of people originating from various communities throughout the wider area and/or their mobile lifestyles? What were the specific living conditions of the population, including dietary habits as well as stress due to disadvantageous health conditions or interpersonal violence? Moreover, do the mtDNA data reflect population-dynamic processes over the course of the establishment and consolidation of the Neolithic on the Iberian Peninsula as well as long-distance contact and migration?

## The Late Neolithic Site at Alto de Reinoso, Spain

**Fig 1:**
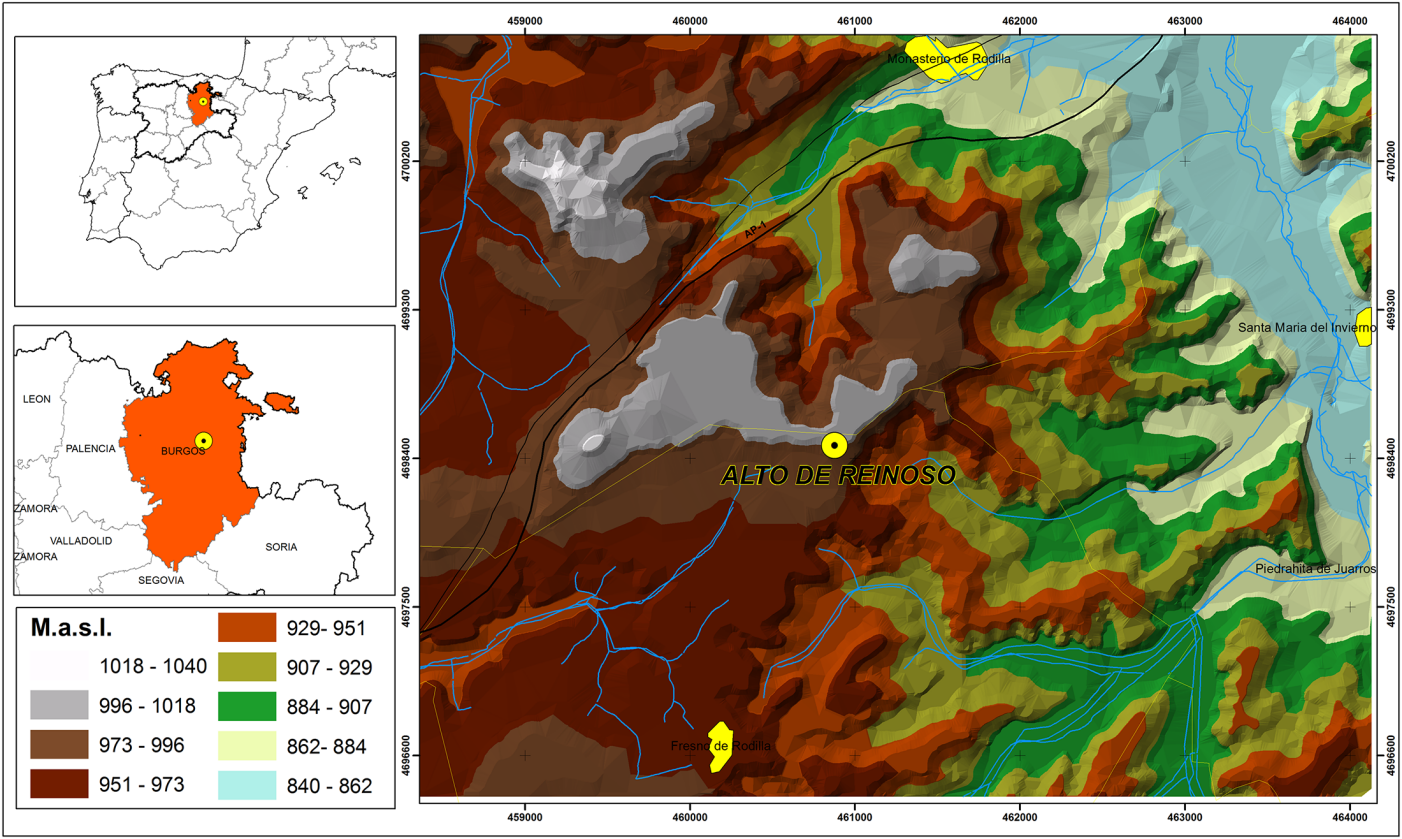
Map showing the geographical location of the Alto de Reinoso megalithic tomb in the Burgos province (Castilla y León, Spain) (graphic: Héctor Arcusa Magallón; Copyright by the authors).

The megalithic tomb at Alto de Reinoso is situated in sprawling hills between the villages of Monasterio de Rodilla to the north and Fresno de Rodilla to the south (Province Burgos, northern Spain). Located on a narrow platform in the Paramo highlands, the burial site dominates the entire surrounding area and is placed in a striking topographical location (Fig 1). The tomb is surrounded by the Northern Meseta landscape, a vast plateau in the interior of the Iberian Peninsula. This plateau has an average elevation of 600 to 700 metres and is flanked by mountain ranges that separate it from the coastal regions: in the north by the Cantabrian Mountains (Cordillera Cantabrica), in the north-east by the Iberian Mountains (Sistema Iberico) and in the south by the Central System. Highly diverse landscapes with strong contrasts in altitude within relatively short distances are characteristic of the Iberian Peninsula [26].

**Fig 2:**
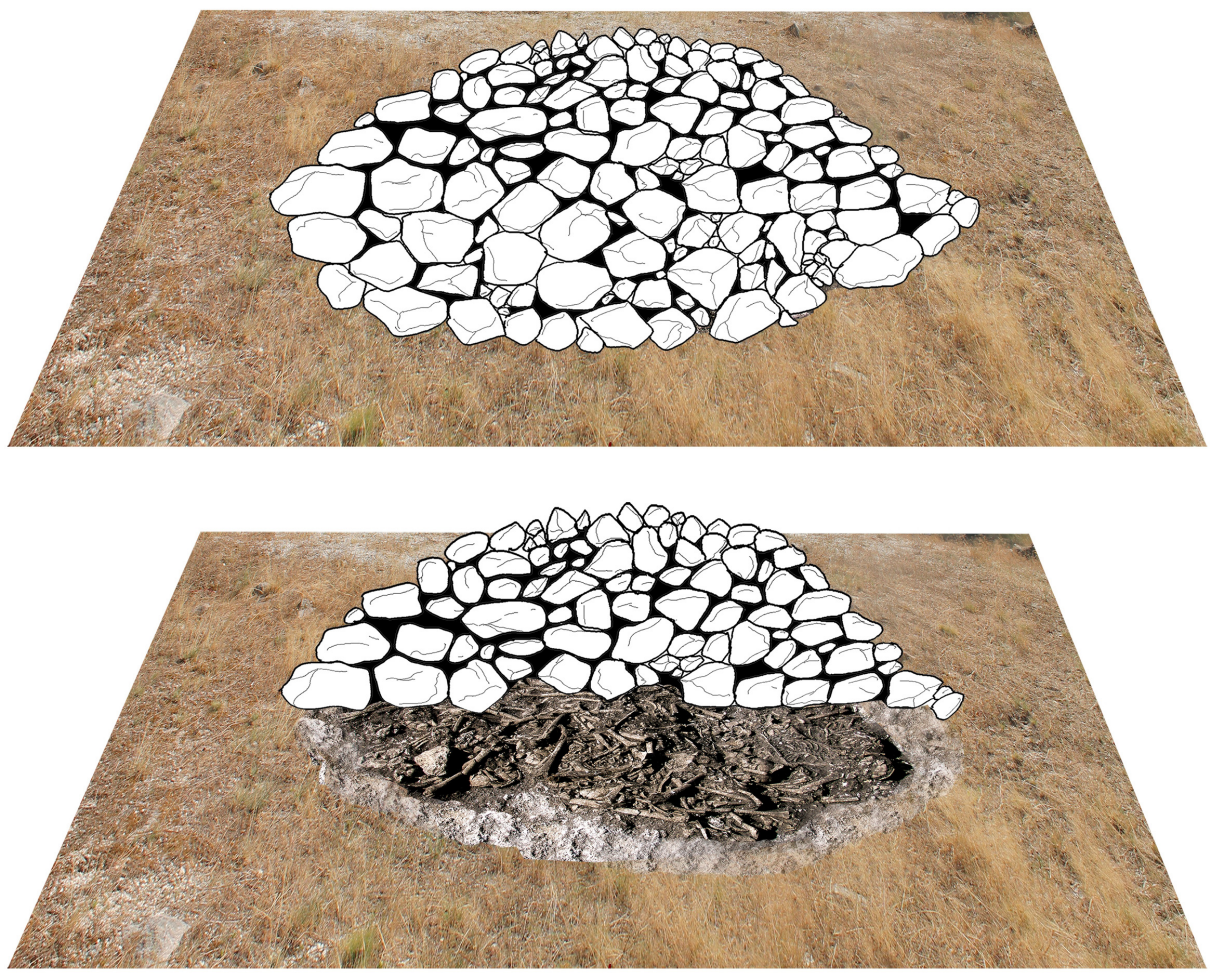
Reconstruction of the original appearance of the megalithic mound of Alto de Reinoso (graphic: Héctor Arcusa Magallón).

The collective tomb had a diameter of approximately three metres and was excavated between 2006 and 2007 by archaeologists from the University of Valladolid, Spain. The primary aim of the rescue excavation was to prevent further destruction of the tomb. Only a few of the stones used in the construction of the mound had been preserved in situ. All the findings made during the excavation suggested that the tomb had originally been some kind of hut constructed in vegetal materials (house of the dead) that was eventually dismantled, closed off and finally turned into a monumental structure by erecting a stone mound above the ossuary (Fig 2).

The excavators identified three distinct layers in the area of the tomb. The uppermost Bronze Age layer, which had been disturbed by the plough during long-term agricultural activity around the grave in more recent times, contained numerous widely scattered stone remains as well as severely disturbed human bones and grave goods. The skeletal remains of two individuals dating from around 1700–1500 cal BC were found in this layer. These two individuals were not included in the study presented here.

The actual Neolithic ossuary containing a surprisingly high density of human bones in two burial layers (top and bottom) of very different states of preservation was discovered beneath this layer. The skeletal remains of the top layer were highly disturbed and included the upper body parts and skull of a male adult (Rein 30) and numerous concentrations of disarticulated human bones: groups of skulls (e.g. the isolated skulls Rein 17 and Rein 23 arranged next to the skull of individual Rein 8; S1 Fig), assemblages of long bones or even skulls intentionally framed by long bones. Most assemblages of skulls and postcranial bones appeared to have been arranged (e.g. Rein 29; see S2 Fig). Furthermore, a huge amount of disarticulated postcranial bones and a number of isolated skeletal parts were found in this layer. Most likely, these were the result of human interference in the context of rituals or were perhaps the result of bone dislocation whilst new bodies were placed in the megalithic tomb.

The bottom layer, on the other hand, revealed complete primary burials in situ, which showed hardly any signs of disturbance. It lay directly on the natural bedrock and contained six relatively complete (Rein 1, 2, 3, 4, 5, and 10) and six half skeletons (Rein 6, 7, 8, 9, 11 and 12) in anatomical order as primary burials (S1 Table and S3 Fig). The individuals had been interred in a crouched position, as it was typical in many Neolithic archaeological contexts [27]: six individuals lay on their left side (Rein 1, 2, 4, 5, 8 and 11) whilst two were found lying on their right side (Rein 3 and 10). However, it was not possible to reconstruct the exact burial sequence of the bodies in the tomb. The skeletal remains had lain relatively undisturbed until being covered by other burials, which provided them with additional protection against relocation. Due to the collective utilisation of the tomb characterised by successive burials whereby older corpses were displaced in favour of more recent ones, individualisation of the human remains was only possible for some of the skeletons. Hence, the reconstruction of the demographic profile of the burial community proved challenging.

The archaeological material recovered from inside the ossuary was not overly abundant and included both personal adornments (stone necklace beads, wild boar tusk pendants) and grave goods (polished stone axes, flint blades and microliths, bone spatulae) (S4 Fig). Some had been intentionally deposited beneath or near the skulls (flint blades and stone polished axes). Two bone spatulae, one of which was decorated, and a stone bead had been placed beneath a skull (Rein 29), and it had also been surrounded by a triangular frame consisting of three long bones (see S2 Fig). Small artefacts such as microliths or beads were easily removed and repositioned along with the bones; one exception to this rule was a lignite bead found on the neck of individual Rein 1. Remarkably, there was a distinct lack of ceramic vessels in megalithic tombs, at least during the earlier period of their use. Researchers assume that this was due to ritual reasons [5]. The archaeological material suggested a date for the megalithic tomb at Alto de Reinoso in the Late Neolithic. Overall the finds exhibited strong chronological coherence and were all artefacts typically found in megalithic tombs throughout the Meseta during the 4^th^ millennium BC.

Radiocarbon dates were obtained from three human bone samples recovered from different layers in the ossuary, giving a maximum chronological range of between 3770 and 3539 (2 sigma cal BC) (S2 Table). The OxCal v4.2.4 software [28] allowed us to narrow down the actual time span represented by the radiocarbon dates to a period of 60 to 80 years, or roughly three generations (S2 Table and S5 Fig). Both the archaeological finds and the radiocarbon dates are characteristic of collective burials from the Late Neolithic [5].

In principle, we may state that collective burials were abundant at the beginning of the 4th millennium BC and exhibited diverse architecture ranging from simple non megalithic barrows, that is to say lacking colossal stone structures, to large passage graves as simple dolmens and allées couvertes [29]. Alto de Reinoso is a good example of a non-megalithic barrow, as the result of the monumentalization of a more basic structure, probably a burial chamber made of wood, mud and other organic materials, the actual shape of which is difficult to ascertain. This structure was finally dismantled as part of a closure ritual, and then marked and monumentalised by erecting a small stone barrow covering the remains of the ossuary [30]. Where a tomb had once stood, a monument to the memory of the ancestors was now erected as a permanent reference to their presence in the symbolic and cultural landscape. Similar examples of this type of monument have been discovered at La Tarayuela (Ambrona Valley, Soria) and at El Castillejo [31].

## Materials and Methods

This study deals with archaeological skeletal material and adheres to the Helsinki Declaration on Ethical Principles for Medical Research Involving Human Subjects. The excavation licenses were issued by the General Director of Cultural Heritage of the Junta de Castilla y Léon (Spain) and are stored in its archives. Following excavation campaigns 2006/2007, the skeletons were transferred to the former Bioarchaeology group at the University of Mainz, Germany for investigation. At present all archaeological remains, including the human bones, recovered at the site of Alto de Reinoso, are stored in the Prehistory and Archaeology lab of the University of Valladolid. The inventory list of the bones in their current storage place is identical to the list supplied in S1 Table of the supplemental information.

A basic concern of anthropological examination of human remains from megalithic monuments is the dense occupation of the tombs both by completely preserved individuals and partial burials as well as isolated bones. One of the preconditions of investigating the demographic structure of a burial community is the analysis of the minimum number of individuals (MNI) which serves as a starting point for all further studies including osteological determination of age and sex, reconstruction of body height, investigation of diseases, trauma and stress markers as well as molecular genetics and isotope analysis. The techniques used in this interdisciplinary integrative study were based on established methods and these are described in the supporting material (S1 Text). A varying number of individuals were available for each section of the examination, depending on the part of the skeleton required to carry out the analysis (Table 1 and S1 Table).

**Table 1:** Results of osteological, mtDNA and isotopic analysis for the human individuals from Alto de Reinoso.

Sample	Ind. / Inv.	Age in yrs	Age Group	Sex	mtDNA haplogroup	^87^Sr/^86^Sr sample	^87^Sr/^86^Sr ± 2 σ (last digit)	δ^13^C‰	δ^15^N‰	Age cal. BC (1σ)
Rein 1	Ind. 1	21–30	Adult	(f)	U5b2b3	Tooth 36	0.70922 ± 3	-19.7	9.7	3698−3657
						Tooth 18	0.70925 ± 3			
Rein 2	Ind. 2	21–30	Adult	(m)	U5b2b3	Tooth 26	0.70932 ± 7	-19.5	9.4	
						Tooth 28	0.70920 ± 3			
						Femur	0.70935 ± 4			
Rein 3	Ind. 3	25–40	Adult	m	U5b3	Tooth 46	0.70957 ± 1	-19.4	10.2	
						Tooth 38	0.70926 ± 2			
						Femur	0.70933 ± 5			
Rein 4	Ind. 4	21–30	Adult	(f)	U5b2b3	Tooth 26	0.70913 ± 3	-19.3	9.7	
						Tooth 17/18	0.70923 ± 4			
Rein 5	Ind. 5	16–20	Juvenile	m	T2b	Tooth 26	0.70910 ± 3	-19.4	9.6	
						Tooth 27	0.70891 ± 2			
Rein 6	Ind. 6	> 21	Adult+	?	V	Pelvis	0.70946 ± 3	-19.5	9.8	
Rein 7	Ind. 7	21–30	Adult	f	-	-	-	-	-	
Rein 8	Ind. 8	41–60	Mature	?	K	-	-	-19.4	9.7	
Rein 9	Ind. 9	30–50	Mature	(f)	V	-	-	-19.4	9.8	
Rein 10	Ind. 10	30–50	Mature	(f)	K	Tooth 16	0.70910 ± 4	-19.6	9.6	
						Tooth 28/38	0.70904 ± 2			
Rein 11	Ind. 11	> 21	Adult+	(m)	-	-	-	-	-	
Rein 12	Ind. 12	21–30	Adult	m	K	-	-	-	-	
Rein 12/2	Inv. 2937	10 ± 2,5	Infans II	-	-	-	-	-19.6	10.1	
Rein 13	near Inv. 1173–74	4–5	Infans I	-	X	Tooth 75	0.70936 ± 5			
						Tooth 46	0.70922 ± 5			
Rein 13/2	Inv. 1173–74	> 21	Adult+	(m)	-	-	-	-19.5	10.3	
Rein 14	Inv. 1171–72	21–30	Adult	(m)	K1a	Tooth 26	0.70907 ± 5	-19.6	10.2	
						Tooth 18	0.70908 ± 5			
Rein 15	Inv. 1149–50	25–40	Adult	?	U5b2b3	Tooth 46	0.70912 ± 6	-19.4	9.7	
						Tooth 48	0.70919 ± 1			
Rein 16	Inv. 1158–60	21–30	Adult	m	X	Tooth 46	0.70920 ± 5	-19.4	10.3	
						Tooth 48	0.70934 ± 7			
Rein 17	Inv. 1022–23	16–20	Juvenile	?	K1a1	Tooth 16	0.70930 ± 2	-19.7	9.6	3691−3636
						Tooth 28	0.70926 ± 2			
Rein 18	Inv. 3102–05	16–20	Juvenile	m	T2b	Tooth 26	0.70909 ± 4	-19.5	10.5	
						Tooth 28	0.70923 ± 2			
Rein 19	Inv. 2867–70	25–40	Adult	(m)	T2b	Tooth 36	0.70945 ± 1	-19.6	10.1	
						Tooth 47	0.70926 ± 4			
Rein 20	Inv. 2835–38	12 ± 2,5	Infans II	-	K1a1	Tooth 36	0.70912 ± 6	-19.4	10.0	
Rein 21	Inv. 1295–98	> 21	Adult+	?	-	-	-	-19.7	9.5	
Rein 22	Inv. 2948–51	12–14	Juvenile	-	U5b	Tooth 46	0.70924 ± 1	-19.5	9.7	
						Tooth 37	0.70912 ± 1			
Rein 23	Inv. 1010–11	16–20	Juvenile	?	U4	Tooth 16	0.70951 ± 1	-19.8	9.6	
						Tooth 18	0.70909 ± 3			
Rein 24	Inv. 2799–801	12–15	Juvenile	-	K1a1	Tooth 46	0.70916 ± 2	-19.2	10.2	
Rein 28	Inv. 1014–15	21–30	Adult	?	H3	-	-	-20.2	9.1	
Rein 28/2	Inv. 1115–16	> 21	Adult+	(m)		-	-	-19.9	9.9	
	near Rein 28/2	2 +/- 8 months	Infans I	-	-	-	-	-	-	
Rein 29	Inv. 2499–501	21–30	Adult	(m)	J	Tooth 26	0.70924 ± 2	-20.0	11.0	3756−3658
						Tooth 28	0.70910 ± 1			
Rein 30	Inv. 381	30–50	Mature	m	T2a1b	Tooth 36	0.70897 ± 2	-20.2	9.4	
						Tooth 37	0.70923 ± 1			
Rein 31	Inv. 1000–02	21–30	Adult	?	U5b	-	-	-20.1	9.5	
Rein 32	Inv. 1265–66	15–17	Juvenile	-	K	-	-	-20.1	10.6	
Rein 33	Inv. 2362–65	16–20	Juvenile	?	X	-	-	-19.9	9.5	
	Inv. 3298–300	11 +/- 2,5	Infans II	-	-	-	-	-	-	
	Inv. 2642–45	8 +/- 2	Infans II	-	-	-	-	-	-	
	Inv. 2317–20	> 21	Adult+	?	-	-	-	-	-	
	Inv. 1600–02	3–4	Infans I	-	-	-	-	-	-	
	Inv. 1310–11	21–30	Adult	?	-	-	-	-	-	
	Inv. 1212–13	> 21	Adult+	(f)	-	-	-	-	-	
	Inv. 809, Inv. 876, Inv. 895	5–6	Infans I	-	-	-	-	-	-	
	Inv. 281	10–11	Infans II	-	-	-	-	-	-	

Ind. = Individual; Inv. = Inventory Number; Infans I = 0–6; Infans II = 7–12; Juvenile = 13–20; Adult = 21–40; Mature = 41–60; Adult+ = not determinded > 21; m = male; (m) = rather male;? = indetermined; f = female; (f) = rather female. Teeth are numbered according to the FDI scheme (Fédération Dentaire Internationale).

In order to reproduce the aDNA results, two or three samples were taken from each of the available individuals (n = 27) for molecular genetics analyses. We generated mitochondrial profiles by analysing the hypervariable regions I and II (HVR I, HVR II) and coding region single nucleotide polymorphisms (SNPs) of the mitochondrial genome (S7, S8 and S9 Tables). Protocols for sample preparations, extraction, amplification setups, and contamination control were applied as described previously [15, 16, 22, 32]. During genetic sample preparation, the tooth enamel—which is not required for molecular analyses—was separated from the dentine and passed on to undergo strontium isotope analysis. This investigation was based on 36 enamel samples from the permanent dentition of 19 individuals. In most cases both an early and a late mineralising tooth were sampled from each individual in order to gain information on potential changes of residence during childhood. Individuals whose bones were too poorly preserved to determine their sex and age at death were excluded from the sampling process. The analytical procedure included the mechanical abrasion of all surfaces and remaining dentine, chemical cleaning with buffered acetic acid, ashing, strontium separation with Eichrom Sr-Spec resin and determination of Sr concentrations with a Quadrupole-Inductively Coupled Plasma-Mass Spectrometer (Q-ICP-MS) and the ^87^Sr/^86^Sr ratios with a Multi Collector-ICP-MS (VG Axiom) at the Curt Engelhorn Centre Archaeometry gGmbH in Mannheim, Germany following the procedures described in Knipper et al. [33]. Details on sample preparation and analyses are given in S1 Text. In addition to the tooth samples, three human bones from the grave and two samples from domestic cattle *(Bos taurus)* also derived from the grave provided comparative data for the interpretation of the ^87^Sr/^86^Sr ratios. During the osteological examination bone samples were also taken for collagen extraction with the aim of analysing the carbon and nitrogen isotope ratios (δ^13^C and δ^15^N) in both humans (n = 29) and animals, including cattle *(Bos taurus)* and a rabbit *(Oryctolagus cuniculus*) (n = 4). The taphonomic alterations of the rabbit bone suggest that it belonged to the same burial context and time period as the human remains. The analytical procedure included mechanical cleaning, demineralization, removal of humic acids, gelatinization, filtration, concentration of the long-chained collagen by ultrafiltration and lyophilisation [22]. The carbon and nitrogen contents were determined by an elemental analyzer (vario EL III, Elementar Analytical Systems) and the isotope ratios by an IsoPrime High Performance isotope ratio mass spectrometer (IRMS; VG Instruments) at the Department of Organic Chemistry at the University of Mainz. More information on sample preparation and analysis is given in S1 Text. The sampled skeletal remains were given laboratory assignations ranging from Rein 1 to Rein 33 (Table 1 and S1 Table). In cases where sampling was not possible the bones were only listed with an inventory number (Inv.). Isolated postcranial bones are not listed, but recorded under its inventory number.

## Results and Discussion

### Osteology

The skeletal remains near the surface in particular bore numerous traces of taphonomic processes including high fragmentation rates, evidence of severe weathering (bleaching, surface changes, micro-cracks, and calcification) as well as root etching. Apart from one exception the absence of gnawing or bite marks on the skeletal remains proves that the dead were quickly placed in the megalithic tomb and that it was inaccessible to carnivores.

#### Minimum number of individuals (MNI)

The minimum number of individuals (MNI) was determined by counting the most abundant parts of the skeletons recovered from the site: crania, pelvis, and femoral diaphysis [34]. Based on right femora and elements of the crania an MNI of 47 can be estimated (S3 Table). However, only 38 near-complete crania or mandibles with teeth were found (21 adults and 17 subadults) which corresponded to a deficit of at least nine adult crania in the burial context compared to the MNI of 30 adults. One possible explanation is that the missing crania were removed during the phase of active utilisation of the burial mound, perhaps in the context of ancestor worship.

#### Sex and age estimation

Sex estimation of human remains is commonly based on morphological traits of the pelvis and cranium or on a metric evaluation following [35] and [36]. At Alto de Reinoso these skeletal elements were only present in individuals from the largely undisturbed bottom layer, so that numerous individuals from the top layer could not be sexed due to poor preservation, and/or due to significant displacement of the bones. The sex estimation of 27 (26 adults, one juvenile) pelvic bones attested to a balanced distribution of both sexes (11 males [40.7%], 12 females [44.4%] and four undetermined adults [14.8%]) (S4 Table). In contrast, apart from a few instances in the bottom layer, the sex estimation of most adult crania was not possible because of poor preservation and high fragmentation. There were more males (n = 9 [42.9%]) than females (n = 5 [24%]) in the sample, although the adults of undetermined sex (n = 7 [33.3%]) could of course include more women than men (S5 Table). Long bones which could be sexed due to a clear affiliation to a sex estimated individual exhibited remarkable sexual dimorphism. It is worth noting that sex determination based solely on crania, without taking into account the pelvis results in an imbalance in the sexes.

In complete skeletons biological age can be expressed either as the dental age or the skeletal age [35–42]. In the case of Alto de Reinoso, at least 30 adults (>20 y) and 17 subadults (≤20 y) had originally been buried in the collective grave [S3 Table]. Crania and mandibles with teeth formed the basis for more precise individual age examinations [S6 Table].

The classification of individuals in different age groups followed Martins’ system [43]. Based on 21 adult and 17 subadult individuals for whom ectocranial suture closure [39] and tooth wear [37;40] or tooth mineralisation [41] could be assessed, twelve individuals [31.5%] were estimated to have been between 21 and 40 years old (age group adult) and four individuals [10.5%] were classified as “mature” (41–60 y). Five further non-infant individuals [13.2%] could not be firmly assigned to either of the age groups. Subadults were represented by four individuals of the age group infans I (0–6 y, [10.5%]), five infans II (7–12 y, [13.2%]), and eight juveniles (13–20 y, [21.1%]). It must be noted in this context that the deposition below ground has a much more significant impact on the preservation of the skeletal remains of infants than on the more robust bones of adults [44], which results in a situation where the bones of some of the children are either not preserved or can at least no longer be individualised. Therefore, the burial community was certainly larger than the MNI would suggest, especially since the individuals of the infans I age group usually comprise the largest proportion of deaths in a prehistoric sample. The deficiency in infants may also have resulted from special burial rites which excluded them from collective burials while older children were interred with the adults [45].

#### Body height

The variation in the adult skeletal morphology and metrics is determined by several factors including health and nutrition during a person’s growth and development, as well as genetics, sex, geography, climate and social conditions. In principle, numerous bones are suitable for the determination of body height [14,46–47]. The long bones of the legs and arms offer the best correlation with a person’s real stature, especially if more than one bone is available for evaluation. Due to the differences in body size between the sexes and between different ethnic groups and populations very different formulae are used in calculating body height [48–52]. Unfortunately, as seen in the other examinations of the skeletons, the state of preservation of the long bones was not very suitable for body height estimation. Taking into account only long bones of clearly sexed individuals, the body height of the males (n = 6) was 159.4 ± 2.0 cm on average (ranging between 156 and 163 cm; [48]). The females (n = 3) exhibited an average body height of 150.0 ± 2.4 cm (ranging between 147 and 152 cm), i.e. nine centimetres shorter than the males. When using the formulae proposed by Breitinger [51] for males and by Bach [52] for females the mean values for both sexes were significantly higher: 162.7 ± 4.8 cm (ranging between 160 and 165 cm) for males and 157.9 ± 4.1 cm (ranging between 154 and 160 cm) for females. Such differences between the sexes are quite common in homogenous populations—seen as larger groups of individuals without subpopulations—where most men are taller than the tallest woman. The low variation within both sexes also suggests that Alto de Reinoso was a closely related community [20]. However, this result must be regarded as an approximation because the total sample size is very small; in seven individuals only one length measurement, mainly of the femur, could be taken into consideration.

#### Palaeopathology of bones and teeth

Substantial surface alterations in the highly fragmented skeletal material were challenging for the diagnosis of diseases, traumata and stress markers. The following description of the pathological findings is therefore not complete with regard to the frequency or the spectrum of pathologies. Indications of pathologies were recorded regardless of whether or not they could be individualised; in cases where it was possible to individualise them, they were listed by inventory number. Overall, the palaeopathological findings were typical of Neolithic farming populations [53–55]. The joints of the spine showed an entire range of degenerative alterations: osteoarthritis (Inv. 3809; Inv. 3589), Schmorl’s nodes (Rein 4), spondylarthrosis and spondylosis (Rein 6, 8, 10, 12, 30) as well as spondylitis (Rein 10). With two fractured ribs (Rein 5, 22), a healed femur fracture (Inv. 1246–47) and a tibia fracture with pseudo-arthrosis (Inv. 1526–27) as well as a hint of blunt force trauma to the cranium (Rein 30), the number of commonly found traumata and violence-related cranial traumata was within the expected low range for the populations of that period [56]. Exceptional enthesiopathies were only diagnosed on the leg bones of Rein 11 and were interpreted as an indicator for more physical activity [57]. Unspecific inflammations were also found in some cases (Inv. 2136–37; Inv. 3383–94; Rein 19; Rein 29; Rein 33). This observation coincides with a general trend showing that the frequency of such lesions increases at the beginning of the Neolithic period [58].

Due to the limitations with regard to individualisation and the large number of isolated teeth it was not possible to determine the frequency of caries (number of affected individuals compared to the total number of individuals). Caries intensity of 14.5% (number of decayed teeth compared to the number of preserved teeth including antemortem tooth loss) among children and adult individuals (loose teeth of unknown individualization are included) was quite low compared to Middle Eastern and European Neolithic populations [55,59**–**60], and especially compared to other Late Neolithic collective burials [25,54,61].

If compared to the contemporary sites at San Juan ante Portam Latinam [62] and Cova del Pantá de Foix [63], the caries intensity of only the permanent dentition at Alto de Reinoso, which was accounted for 14.3% (including antemortem tooth loss), fits in well with 13.2% at San Juan Portam Latinam and 15.3% at Cova del Pantá de Foix, although it is unclear whether antemortem tooth loss was taken into account for Cova del Pantá de Foix or not.

However, the unusually high caries intensity in deciduous teeth (17.8%) suggests that nearly all subadults suffered from tooth decay (exclusively molars) in their primary dentition. With 13.4% affected deciduous teeth similar values exist at San Juan ante Portam Latinam [62] while the collective grave of Benzingerode, Germany, did not contain a single carious deciduous tooth [25]. Several datasets on caries intensity in deciduous teeth in earlier Linearbandkeramik communities in Germany exist: 5.4% at Aiterhofen [64] and 2.7% at Wandersleben [65]. The reasons for the rather high percentages of caries in deciduous teeth seem to be associated less with the socioeconomic status of the population but rather with specific dietary habits, especially the consumption of carbohydrate-rich sticky food such as porridge but also due to prolonged breastfeeding [66,67].

With regard to stress markers, enamel hypoplasia [66,68] was observed in 11.8% of the permanent teeth (canines in particular) and in 10.2% of the deciduous teeth (molars in particular), although the poor state of preservation did not allow for an individualised analysis. When comparing the incidence of enamel hypoplasia in the permanent dentition at Alto de Reinoso with 36.5% at Cova del Pantá de Foix, significantly fewer teeth showed enamel hypoplasia at Reinoso. A similar comparison between the deciduous teeth was not possible. Studies from the Portuguese collective tombs at Feteira II, Cova da Moura, Paimogo I and Cabeço da Arruda I [69], which dated from the Neolithic to the Chalcolithic, revealed less enamel hypoplasia than was observed at Alto de Reinoso but were based on small sample sizes. Cribra orbitalia could not be analysed due to considerably altered cranial bone surfaces and severe crushing of the crania.

### Molecular genetics

**Fig 3:**
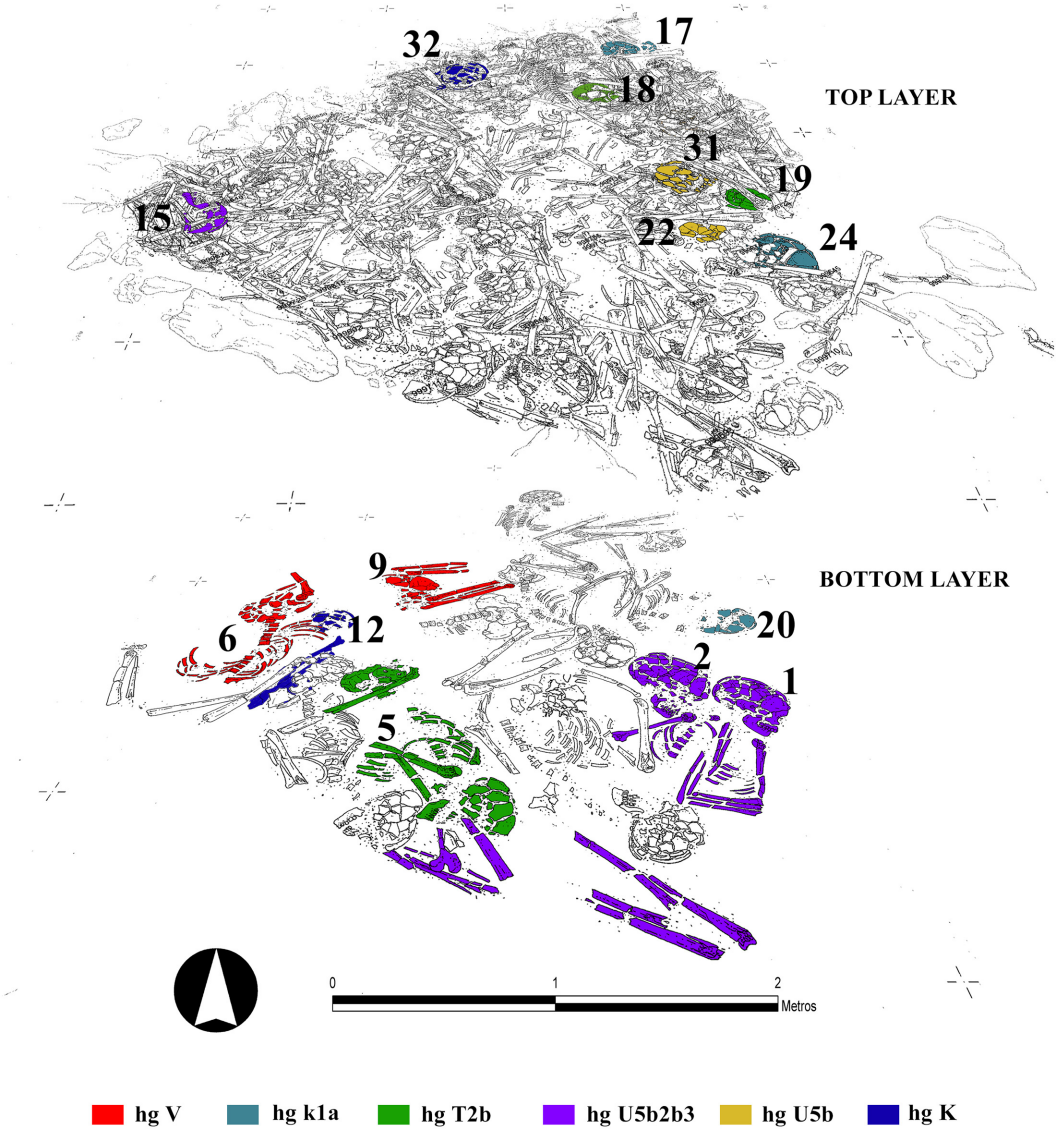
Superposition of different layers of the Neolithic ossuary indicating the individuals with the same genetic profile (graphic: Héctor Arcusa Magallón).

The HVR I and coding region single nucleotide polymorphisms (SNPs) of the mitochondrial genome (GenoCoRe22; [32]) were successfully reproduced for 26 out of the 27 individuals analysed (www.ncbi.nlm.nih.gov/genbank; accession numbers KT868907-KT868932), resulting in an extremely high amplification success for ancient DNA analyses especially in relation to studies from south-western Europe (S1 and S7 Tables). Furthermore, H –plex analyses [70] defined the specific subhaplogroup of an individual belonging to haplogroup H (S8 Table). The individuals buried in the megalithic tomb at Alto de Reinoso belonged to mtDNA (sub-) haplogroups H3 (n = 1; 3.7%), J (n = 1; 3.7%), K (n = 8; 29.6%), T2 (n = 4; 14.8%), U4 (n = 1; 3.7%), U5b (n = 7; 25.9%), V (n = 2; 7.4%), and X (n = 3; 11.1%). The dominance of haplogroups K, T2 and U5b in particular can be ascribed to the large number of shared lineages among the individuals buried in the tomb. More than half of all individuals analysed have characteristics in their mitochondrial profile that match one or two of the other individuals, pointing to maternal relations within the burial community. Shared lineages for all markers analysed could be found in individuals Rein 17, 20 and probably 24 [K], in Rein 5, 18, and 19 [T2b], between Rein 1, 15, and potentially 2 [U5b3], and also between Rein 12 and 32 [K1a], Rein 22 and 31 [U5b], and Rein 6 and 9 [V] (S1 Table). With reference to the layers in the tomb, shared lineages can be observed between individuals from the same layer (e.g., Rein 6 and 9; Rein 2 and 1 [bottom layer]; Rein 17 and 24; Rein 22 and 31; Rein 18 and 19 [top layer]), but also in individuals found in different layers (e.g., Rein 1 and 2 with Rein 15, Rein 5 with Rein 18 and 19 and Rein 17 and 24 with Rein 20; Fig 3). All in all 17 mitochondrial links were identified among the 26 individuals.

**Fig 4:**
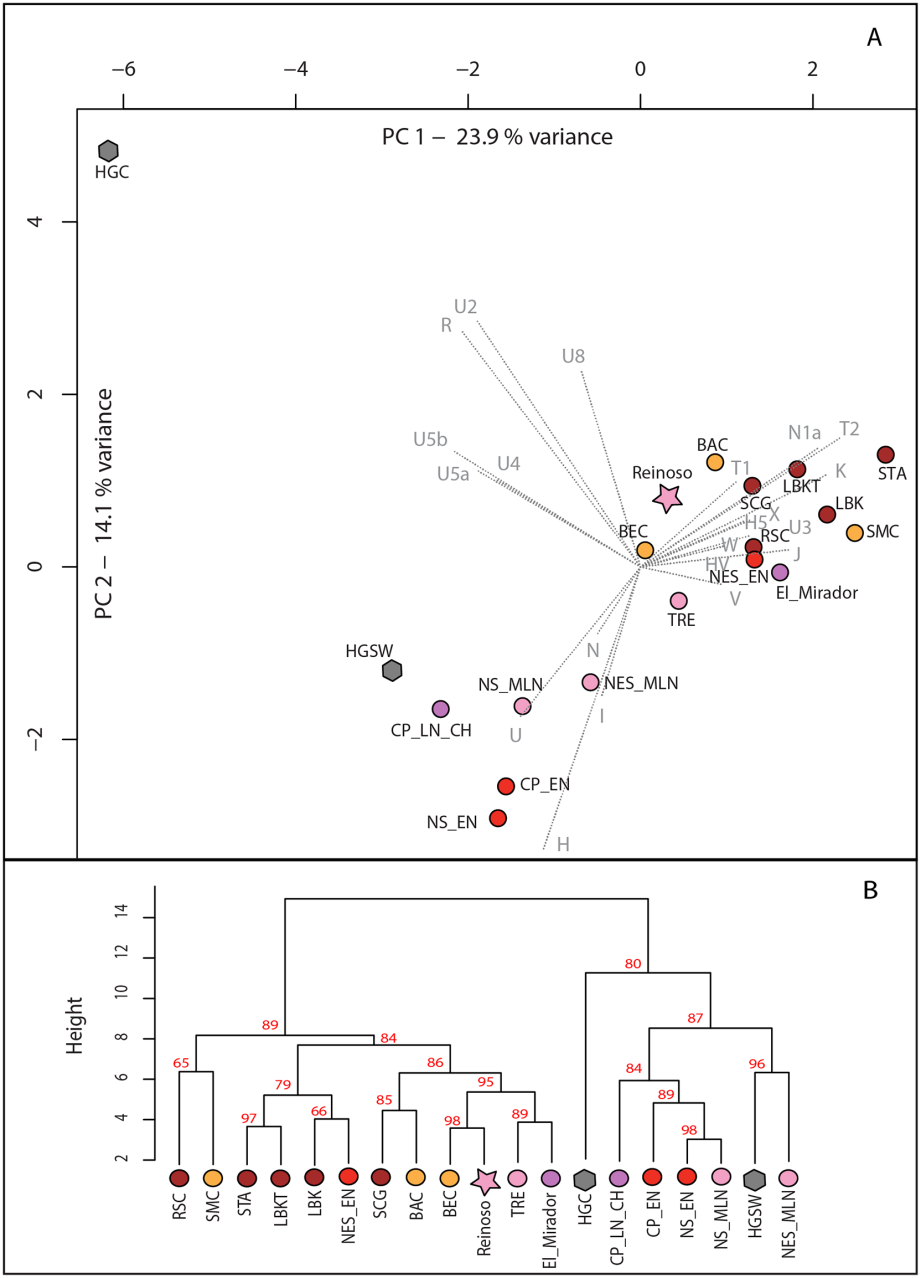
Principal component analysis (A) and Ward type hierarchical clustering (B) with 19 Western and Central European populations. On both image parts south-western European hunter-gatherers [HGSW] and Central European hunter-gatherers [HGC] are symbolised by grey hexagons, Early Neolithic Iberian groups (Central Portugal [CP_EN], north Spain [NS_EN], and northeast Spain [NE_EN]) by red circles and Middle/Late Neolithic groups by pink star and circles (Alto de Reinoso [Reinoso], north Spain [NS_MLN], northeast Spain [NES_MLN], south French Treilles group [TRE]). Magenta-coloured circles indicate the chalcolithic El Mirador cave [El_Mirador] and Late Neolithic-Chalcolithic Central Portugal [CP_LN_CH]. Early Neolithic groups in Central Europe and Carpathian Basin are represented by brown circles (Starcevo culture [STA], Linear pottery culture in Transdanubia [LBKT], Linear Pottery culture in Central Europe [LBK], Rössen culture [RSC], Schöningen group [SCG]), Middle Neolithic groups in Central Europe (Salzmünde Culture [SMC], Baalberge Culture [BAC], Bernburg Culture [BEC]) are in orange. (B) Cluster significance is given as AU (Approximately Unbiased) p-value in %, which is computed by multi-scale bootstrap resampling with 10,000 replicates (graphic: A. Szécsényi-Nagy).

In order to investigate genetic connections between the Alto de Reinoso site and other 18 prehistoric European populations, comparisons were made with published data from elsewhere in West Europe on one hand [71–80], and from nine prehistoric Central European groups on the other [16,32,81–89] by means of a principle component analysis (PCA) and Ward type hierarchical clustering (Fig 4a and 4b). The first and second principal components (PCs) account for approximately 38% of the overall variability of the dataset on the PCA. The closest groups to Alto de Reinoso are the Early/Middle Neolithic Bernburg (BEC) populations in Central Europe, the Late Neolithic south French Treilles group (TRE) and the Chalcolithic El Mirador community (Fig 4b). The north Spanish and the central Portugal Neolithic groups together with the south-western European hunter-gatherers (HGSW) are separated from the main cluster along the N, U, and H haplogroups. The Central European hunter-gatherer metapopulation (HGC) is clearly separated from the Iberian groups and Central European Neolithic too, indicating population discontinuity during Neolithisation of Central Europe [16,86,89].

Alto de Reinoso and the Early Neolithic population in north-eastern Spain appear to consist of a mixture of haplogroups typical of other Neolithic populations in Spain and Portugal that are mainly characterised by haplogroups H, N and U5b, and haplogroups V, J, K, N1a, T2, and X that are typical for Early and Middle Neolithic groups in Central Europe. However, the PCA reveals a separation between the Early/Middle Central European cluster (including Alto de Reinoso) and the cluster composed of the Neolithic-Chalcolithic Portugal, Neolithic north Spain, and the north-eastern Spanish Middle Neolithic population, where the latter cluster shows marked mixture with populations of hunter-gatherer legacy. Furthermore the PCA indicates common traits between the Central European Early/Middle Neolithic genetic substrate, the Early Neolithic population of northeastern Spain, the Late Neolithic Reinoso and Treilles community and the population of the Chalcolithic El Mirador site, probably originated from a common Near Eastern ancestry.

### Residential mobility

**Fig 5:**
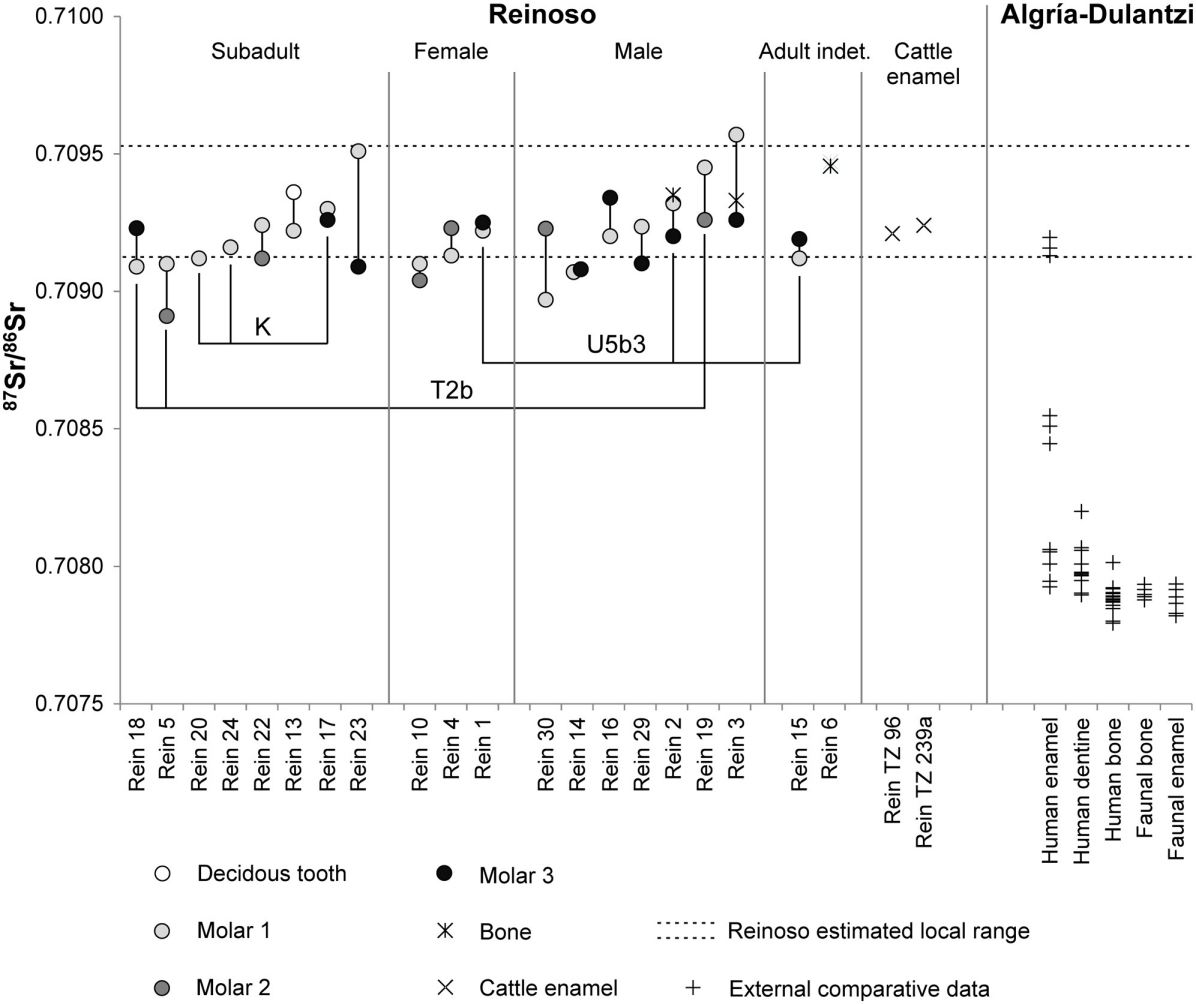
^87^Sr/^86^Sr ratios of human and faunal enamel and bone samples from Alto de Reinoso and from Algría-Dulantzi in comparison. The data from Reinoso are grouped by age and sex and sorted by the ^87^Sr/^86^Sr ratios of deciduous teeth and first molars within each group. The dashed lines illustrate the isotope ratios of the local biologically available strontium in human bone and cattle enamel as calculated from the average plus and minus two standard deviations. (Data for Algría-Dulantzi [92]) (graphic: C. Knipper).

The ^87^Sr/^86^Sr ratios of a total of 36 human enamel samples (s) from 19 individuals (n) varied between 0.70893 and 0.70948 (mean: 0.70920 ± 0.00027; 2 σ) (Fig 5 and S1 Table). The ranges of the adults of both sexes and the subadults largely overlapped. Most likely due to the small sample size the data of the adult females (0.70899–0.70933, n = 3, s = 6) appeared less variable than those of the subadults (0.70891–0.70951, n = 8, s = 14) and the adult males (0.70897–0.70957, n = 7, s = 14). Indications pointing to local baseline values at the site itself came from three human bones (0.70924–0.70952) and two samples of domestic cattle enamel (0.70918–0.70927) (S10 Table). Their average, at two standard deviations, exhibited a range of between 0.70912 and 0.70952. The teeth of a number of individuals had ^87^Sr/^86^Sr ratios just below the lower limit (Rein 5, 10, 14, 15, 18, 23, 29, 30) or above the upper limit of this range (Rein 3). However, because the number of comparative samples was very small and may lead us to underestimate the variation of the local baseline values, these numbers provide no clear indication with regard to non-local individuals. Moreover, the overall data distribution of the human enamel was continuous and lacked any results that clearly diverged from the main data cluster.

Nevertheless it is worth mentioning that in addition to the ^87^Sr/^86^Sr ratios of their first molars beyond the local baseline range, the differences between the early and the later forming teeth of Rein 23 (Δ^87^Sr/^86^Sr_M1-M3_ = 0.00042), Rein 3 (Δ^87^Sr/^86^Sr_M1-M3_ = 0.00031), Rein 30 (Δ^87^Sr/^86^Sr_M1-M2_ = 0.00026), and Rein 5 (Δ^87^Sr/^86^Sr_M1-M2_ = 0.00019) were larger than those of all individuals with both measurements within the local range. As these differences are larger than the analytical error they may point to a shift in diet catchment areas during childhood, even though it would have involved localities with very similar geological conditions to those at Alto de Reinoso. The site is surrounded by calcarenites and conglomerates of the Lower Neogene [90], which were also used in the construction of the tomb itself and are widely distributed around the site (S6 Fig).

Only a few studies on bioavailable ^87^Sr/^86^Sr isotope ratios from archaeological sites on the Iberian Peninsula have been published so far [91**–**93]. A rich comparative dataset from the area, which includes human enamel, bone, dentine, and faunal samples was gathered at the early medieval cemetery of San Martín de Dulantzi (Algría-Dulantzi, Álava) some 90 km north-east of Reinoso, where Cretaceous limestone and marls prevail [92]. This study illustrates the existence of localities with less radiogenic baseline values than those observed at Reinoso and the possibility of identifying distinctly non-local individuals in northern Spanish burial contexts.

### Dietary reconstruction

**Fig 6:**
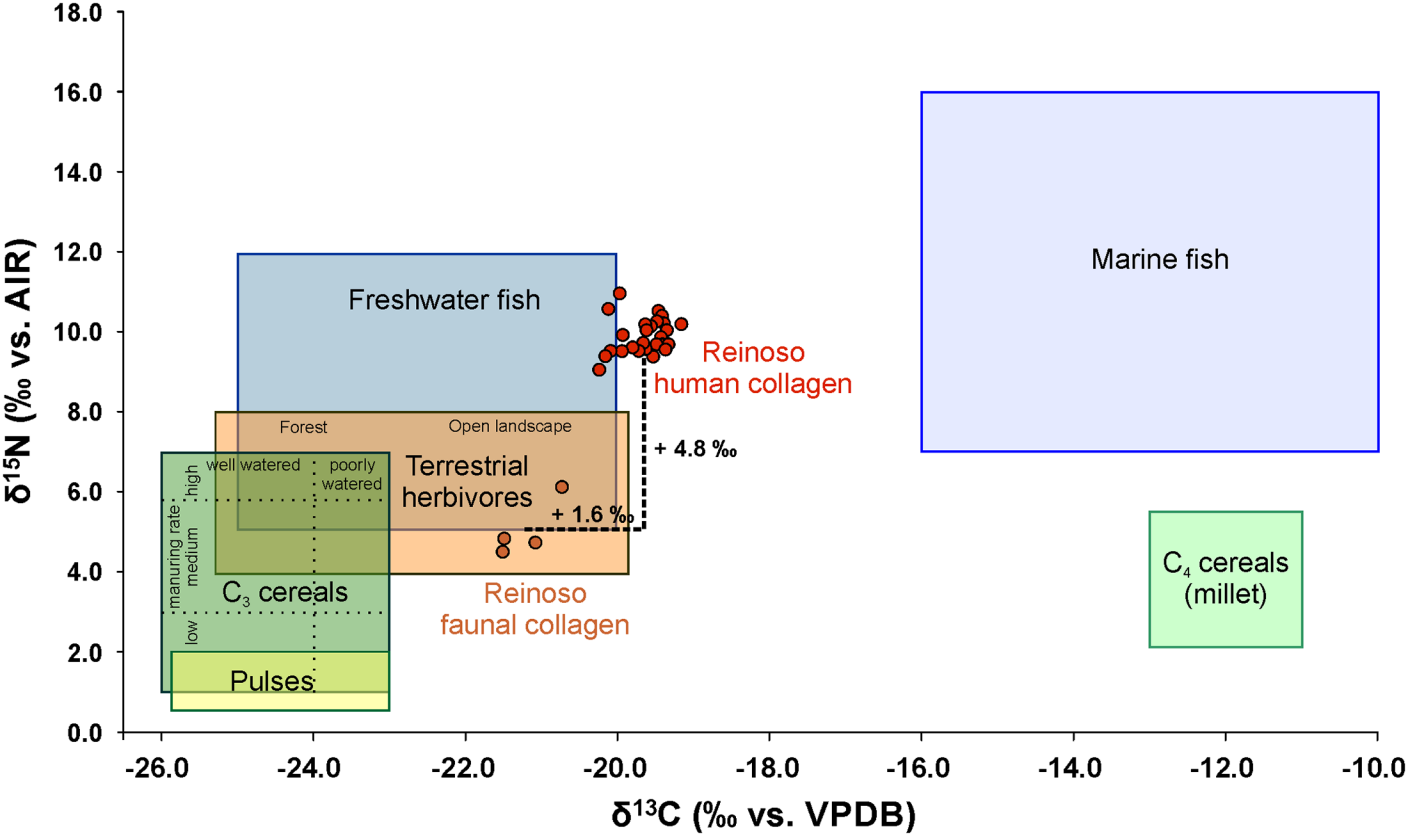
Carbon and nitrogen isotope ratios of the human and faunal samples from Alto de Reinoso in comparison to the isotope ratios of potential major food sources. Comparative data are taken from [107–108, 135, 144–146] (graphic: C. Knipper).

**Fig 7:**
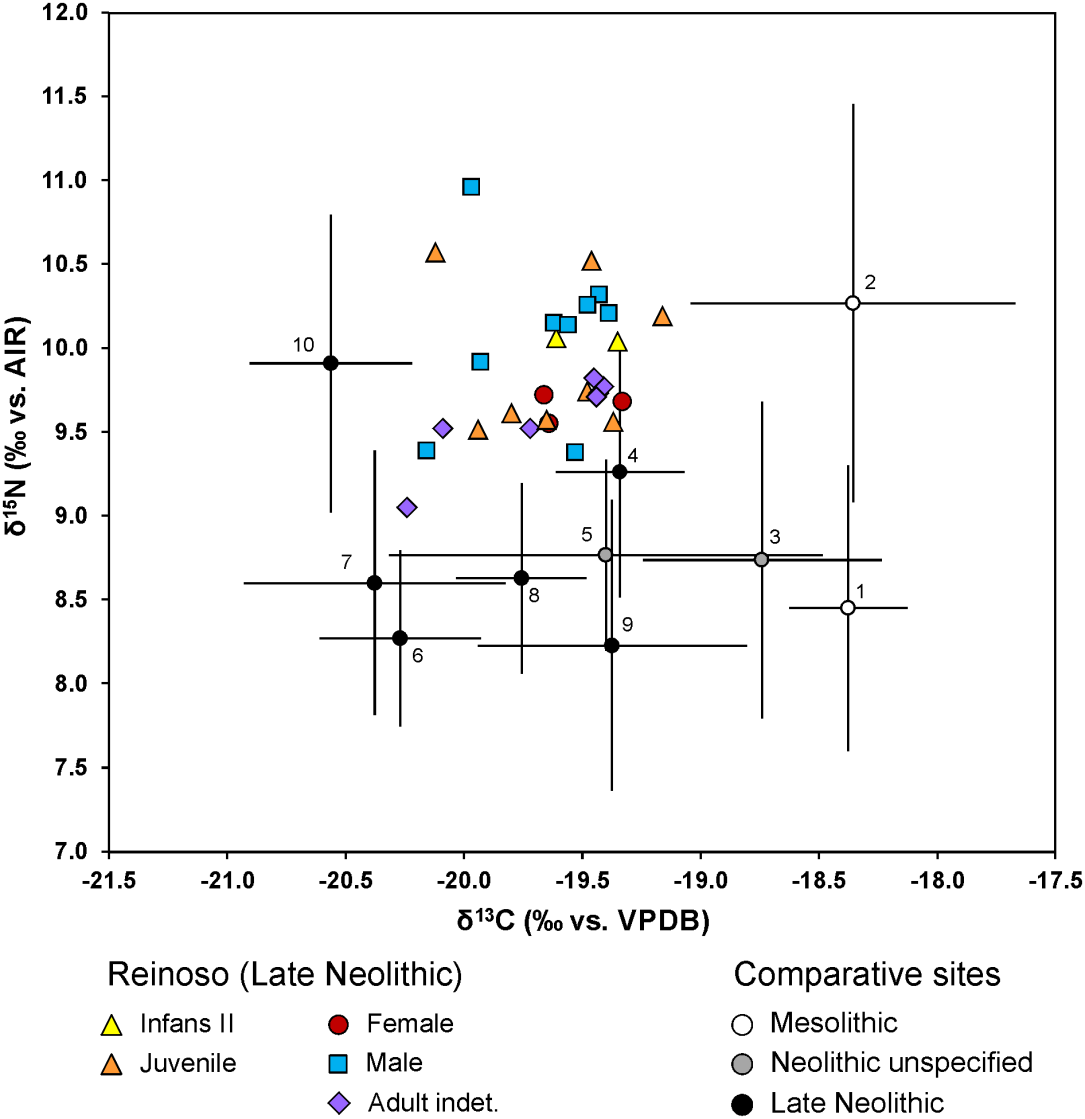
Scatter plot of δ^13^C and δ^15^N values and faunal collagen data from the Alto de Reinoso collective tomb and data from comparable Mesolithic and Neolithic sites. 1: Valencia: Santa Maria, Penya del Camtador, Clingel des Mans Nou (Spain [104]), 2: El Collado (Spain [137), 3: Costamar (Spain [143]), 4: Cova de la Pastore (Spain [139]), 5: Mean values from Casa da Moura, Gruta do Caldeirao, Gruta da Feteira, Gruta da Fontainhas, Roche Forte II, Cerca do Zambujal, Melides (Portugal [141]); 6: Fateira II (Portugal [69]), 7: Paimogo I (Portugal [69]), 8: Cabeco da Arruda (Portugal [69]), 9: Languedoc area, Les Crés, Montou, (France [138]), 10: Garonne area, Cugnaux, Villeneuve-Tolosane, Narbons (France [138]) (graphic: C. Knipper).

The results of the stable carbon and nitrogen isotope analyses of the human and animal bones are presented in Table 1, S1 and S10 Tables. Most of the human bones and four animal samples (three cattle, one rabbit) analysed met the quality indicators for collagen. Four human samples yielded less than 1% of collagen. Because the other quality criteria (C %, N %, and C:N ratio) of these samples were in the accepted ranges [94–96] the samples were included in our considerations. Their lower collagen yields are likely to have resulted from a substantial loss of higher degraded collagen during the ultra-filtration step [97]. The human stable isotope ratios ranged from -20.2 to -19.2 ‰ (average -19.6 ± 0.3 ‰) for δ^13^C and from 9.1 to 11 ‰ (average 9.9 ± 0.4 ‰) for δ^15^N (Figs 6 and 7). The average values of all adults were -19.7 ± 0.3 ‰ in δ^13^C and 9.8 ± 0.4 ‰ in δ^15^N (n = 19). The δ^13^C mean ratios for males were -19.7 ± 0.3 ‰ (n = 9) and those for females -19.5 ± 0.2 ‰ (n = 3). The δ^15^N mean values of the males (10.1 ± 0.5 ‰) were slightly higher than those of the females (9.7 ± 0.1 ‰). The stable isotope ratios from the faunal samples varied from -21.5 to -20.7 ‰ for δ^13^C (average -21.2 ± 0.4 ‰) and from 4.5 to 6.1 ‰ for δ^15^N (average 5.1 ± 0.7 ‰) (Figs 6 and 7 and S10 Table). The data for the different human age groups are presented in Fig 7. All the groups tested were normally distributed (Kolmogorov-Smirnov-test for normal distribution) and an analysis using the ANOVA and Post-Hoc Tuckey HSD-tests showed no significant differences between the sexes or between the ages.

The stable isotope data of the humans from Alto de Reinoso showed values that are typical of a mixed diet in a food web based on C_3_ plants. There is no indication for substantial millet or marine diet intake, which would both have resulted in higher δ^13^C values. The latter is not surprising considering the location of the site in the Meseta [98].

To reveal better information on the protein sources we calculated the difference between the faunal and human values. The average offsets between cattle and humans were +1.7 ‰ in δ^13^C and +5.2 ‰ in δ^15^N. Both the differences in the carbon and in the nitrogen values were higher than the enrichment typical of one trophic level, which is 1–2 ‰ for carbon and 3–5 ‰ for nitrogen [99–103]. This does not exclude the consumption of beef, but suggests that foodstuffs with higher δ^15^N values have contributed substantially to the human diet. The differences between the isotope ratios of the rabbit collagen and the average of the humans were +1.1 ‰ in δ^13^C and +3.7 ‰ in δ^15^N. These numbers are closer to the normal trophic level values even though the overall context makes it unlikely that rabbits contributed largely to the animal protein supply.

Because cattle are unlikely a primary source of animal protein, other animals with higher δ^13^C and δ^15^N values such as pig, goat and sheep and their dairy products as well as possibly also freshwater fish should be considered. However, samples of these species were not available in the megalithic tomb at Alto de Reinoso. Therefore, we drew on analyses from other sites on the Iberian Peninsula, even though their explanatory power for our data was perhaps limited due to spatial and chronological differences. Collagen of wild boars from the Mesolithic site at Clingel del Mas Nou showed δ^13^C values of -19.9 ± 1.1 ‰ and δ^15^N values of 6.6 ± 1.0 ‰ (n = 6) [104]. Pigs from Late Neolithic sites in Portugal with -20.5 ± 0.6 ‰ δ^13^C and 7.1 ± 2.3 ‰ δ^15^N (n = 8) revealed even higher nitrogen isotope values. The ovicaprine collagen from Late Neolithic Portuguese sites showed mean values of -20.7 ± 0.3 ‰ δ^13^C and 5.6 ± 0.4 ‰ δ^15^N [69]. The data ranges of these samples fit in better with the isotope ratios of animals that could have contributed protein to the human diet consumed at Reinoso. Moreover, archaeological findings suggest that goat and sheep were the most important domestic animals in Spain during the Early and Middle Neolithic [105–106]. Goats and sheep could have been among the most essential basic foods consumed at Reinoso. Moreover, consumption of freshwater fish is possible, but due to their often very variable stable isotope data [107**–**108] hard to estimate without contemporary comparative samples from the Meseta.

Durum wheat, hulled and naked barley and faba beans were probably the main sources of plant protein [109**–**110]. Aguilera and colleagues [109] analysed the nitrogen isotope compositions of naked wheat and naked barley recovered from the Los Castillejos site which dated from between 4000 and 2500 BC. The nitrogen isotope values of barley of between 4 and 6 ‰ remained comparatively stable over time, while the wheat varied more, from 2 to 7 ‰. The nitrogen isotope values of plants can be strongly influenced by fertilising and by arid climate conditions [111]. The data published by Aguilera and colleagues [109] clearly indicate that it is essential to consider plant protein data when examining the dietary habits of the Alto de Reinoso population. Crop plants provided a mean nitrogen isotope value of 4.8 ‰ which is highly similar to the mean nitrogen values of the cattle. It is therefore hardly possible to distinguish between plant and animal derived protein in the diet.

## Integrative Analysis and Interpretation

Because of its remote elevated location (Meseta) the economy at Alto de Reinoso would have been characterised by small-scale farming and herding. The economic subsistence and the remote geographical location would obviously be reflected in the results. Moreover, the short period of use of the tomb and the preservation of the skeletal remains, which was of a sufficient quality for most of the analyses carried out, provided an opportunity to generate scientific data that would allow us to ascertain whether the subsistence conditions had had an impact on the social structure within the community, what it could have looked like and what dynamics may have developed. Besides the standard osteological methods to reconstruct the fundamental “biographical” data, this approach requires the use of modern methods of analysis, several of which were in fact employed in this study.

### Megalithic tombs—human connections in life and in death

How prehistoric communities dealt with death is reflected in predetermined communal rituals. These also incorporated the place where the dead were laid to rest. The Neolithic megalithic tombs in Europe represent a particular type of collective burial. Compared to individual graves in a cemetery, the communal use of a “house of the dead” more clearly illustrates the bond within a community where the individual is not at the forefront, in life or in death [112]. It was not until the Chalcolithic period and the Bronze Age that social differentiation increased and individuals and groups came to the fore more and more clearly [113].

According to the archaeological finds and features as well as the radiocarbon dates the collective tomb was used by a small farming community for a period of three generations at most (generation = 25 years). This rather short period of use appears typical of the megalithic ossuaries in the Iberian interior, as seen at La Tarayuela, La Velilla, La Sima (especially phase II), La Vega and Los Zumacales [5]; all these tombs were used by only a few generations (no more than four). Given the estimated period of use, the MNI calculated at 47 for Alto de Reinoso suggests that an average of one or two people died every year; this number would include infants, which were largely absent. The MNI is within the range typically observed at Neolithic megalithic tombs situated relatively close to Alto de Reinoso such as La Peña de la Abuela [114], La Sima [115] and La Tarayuela [116], and also at other Spanish sites further afield such as San Juan ante Portam Latinam [62] in the Basque country, Cerro Virtud in Almeria [117] and Cueva de Malalmuerzo in Andalucia [118] and at Late Neolithic sites in Portugal [119]. All the data and findings generated at Alto de Reinoso in relation to the tomb, its size, the number of burials and its period of use attest to relatively uniform funerary rites at that time in the Meseta and (in some cases far) beyond.

### Is the demographic composition of the community representative?

From the point of view of palaeodemographics a skeletal sample is considered representative if the adults exhibit a natural stratification regarding age and sex distribution and if the proportion of infants is not below 20% due to the high mortality rates of children under 1 year [120**–**121]. Because the data gathered at Alto de Reinoso did not fulfil these criteria, the sample would at first glance not be deemed representative. The percentages of adults and subadults at Alto de Reinoso were offset in favour of the adults because the infants and small children in particular were clearly underrepresented (S3 Table). The lack of bones of the age group infans I can generally be attributed to taphonomic processes, particularly in collective burials, where a lot of disturbance occurs over their period of use. At Alto de Reinoso the group was represented by individual bones but could not be accounted for as a whole. In a comparative study on the demographic structures in megalithic graves in northern Spain Fernández-Crespo and de-la-Rúa [122] presented results similar to those found at Alto de Reinoso with a shortage of children less than five years, although they also pointed to examples of a lack of mature and senile adults. However, compared to Cerro Virtud [117], La Peña de la Abuela [114], La Tarayuela [116] and La Sima [114] the tomb at Alto de Reinoso contained more children and adolescents, whilst yielding fewer compared to San Juan ante Portam Latinam [62] and Cueva de Malalmuerzo [118].

The age determinations yielded mainly adolescents (n = 8) and young adults (n = 12), whilst older adults (> 41 y) were underrepresented. However, taking into account the adults whose ages could not be ascertained (n = 7) could potentially increase the number of mature individuals. The ratio of sexes which favoured the males (determination based on crania) was put into perspective by the examinations of the pelvic bones which indicated a rather more equal distribution (S4 Table). Therefore, the predominance of males in megalithic graves as has been suggested due to comparable data gathered at Cerro Virtud [117], La Peña de la Abuela [114], La Tarayuela [116] and San Juan ante Portam Latinam [62] was not observed at Alto de Reinoso. The idea of the predominance of males was already discredited elsewhere [122]. Based on the Las Arnillas dolmen where more males than females were identified Delibes de Castro argued for a possible social relegation of women [123]. He believed that, besides problems linked to pregnancy and childbirth, women may have had a lower life expectancy due to social discrimination, which could perhaps even have impacted on their diet.

The striking lack of skulls can again be explained by taphonomic processes. Some of them were found as part of groupings with isolated long bones elsewhere in the tomb which, however, does not explain the shortfall. We would suspect that skulls were removed from the tomb, perhaps as part of ritual acts within the context of ancestor worship. The fact that children and young adults would not be considered ancestors could explain the relative underrepresentation of older individuals in the tomb. Numerous examples of ancestor worship using skulls in *pars pro toto* ceremonies are known from the Neolithic period [124**–**128].

### The structure and dynamics of the community at Alto de Reinoso

The best way to answer questions regarding the social structure and potential dynamics within the Alto de Reinoso community, beyond the demographic data, is to study the molecular profiles. Taking into account the MNI, the number of burials in the two layers and its period of use, the bottom layer probably contained one generation, whilst the top layer is more likely to have included members of two generations. This means that all individuals from the same layer were separated by one generation at most. Reproducible genetic profiles were generated for 9 individuals from the bottom layer and 17 individuals from the top layer. The fact that, according to the molecular genetic analyses, 15 of the individuals buried at Alto de Reinoso shared their mtDNA haplotypes with at least one other person is not surprising given the limited size of the community, nor should it come as a surprise that pairs of individuals with identical haplotypes were found buried directly beside each other in the less disturbed part of the tomb, which suggests very close genetic relations (Fig 3). The fact that shared maternal lineages generally occurred between individuals within both layers and also between individuals from different layers is population-biological evidence for a certain degree of continuity within the group. On the other hand, some of the genetic profiles only occurred in one of the layers.

One could argue that the lineages that were especially frequent at Alto de Reinoso—mainly of haplogroups K and T2—were also identified at other Neolithic sites in Spain (Cova d´Avellaner, Can Sadurní, San Pau de Camp, and Chaves; [74,77]) and therefore perhaps simply reflect common Neolithic lineages instead of mitochondrial kinship. However, these lineages were not identified as frequently by any of the prehistoric aDNA studies carried out elsewhere and the rCRS lineage of haplogroup V (Rein 6 and 9) was exclusively found at Alto de Reinoso. The fact that a total of six different mitochondrial lineages occur in two or three individuals argues against random haplotype matches and for the existence of maternal kinship among the individuals buried at Alto de Reinoso. Given the period of use of the tomb one would not expect more than two or three members of any particular nuclear family to have been buried in the tomb, and these may represent successive generations. In summary, the overall picture is that of a closely related community that did not experience any noteworthy changes over the period of time the collective tomb was in use.

The absence of dynamic changes within the group is further confirmed by the ^87^Sr/^86^Sr isotope ratios. There was no clear evidence pointing to non-local individuals from distinctly different geological areas. In examining the dataset as a whole, one of the striking features is that three of four individuals with a slightly divergent Sr isotope pattern compared to the remainder of the population (Rein 3, 23 and 30), had genetic profiles that did not have any parallels in the collective burial (Fig 5), despite the fact that numerous haplotypes were represented at the site. The lack of evidence regarding to biological relations between these three individuals and the other members of the community supports the assumption that they had come from elsewhere. The isotope ratios in the late mineralising tooth crowns of Rein 3 and Rein 30 were equivalent to the local baseline, which means that if there had been a change of location it would have occurred during their childhood.

The individuals with identical haplotypes, on the other hand, largely yielded Sr isotope ratios typical of the locality and very slight differences between the ^87^Sr/^86^Sr ratios in the earlier and later mineralised teeth, which both argues against residential changes. This further confirms the notion of a closely related community, several generations of which sourced their food from geologically uniform economic areas. One possible exception was the 16–20 year-old male individual Rein 5 (hg T2b) with ^87^Sr/^86^Sr ratios typical of the locality in the first permanent molar, whilst the second permanent molar yielded the lowest Sr isotope ratio of the entire dataset. Whilst it is quite possible that the individual was born locally, he may have lived with a different community for some of his childhood. However, due to the only slight difference in the ^87^Sr/^86^Sr ratios of both teeth and the small number of comparable samples it must be emphasised that this is only one possible interpretation. The Sr isotope ratios overall paint a picture of the funerary site at Alto de Reinoso as a largely closed community within which (long-distance) mobility probably played a rather insignificant role. Because the sample only included a small number of females, no definitive conclusions could be drawn with regard to the rules of residence (matri- or patrilocality).

### Alto de Reinoso in view of the Western and Central European Neolithic population and settlement history

It is widely accepted that the Neolithic spread to the Iberian Peninsula from the Near East via the Mediterranean route [129]. Dependent on the Mesolithic background, there is, however, considerable variation with regard to the acceptance of pioneer farmers and the assimilation of autochthonous hunter-gatherers in different areas of Spain and Portugal. Because Western and Central Europe differ both in the beginning of the Neolithic and in the admixture of the first farmers, it is essential to analyze sites from various regions [86]. General statements concerning the settlement and population history of Europe can only be reached in comparison.

When compared to other Neolithic groups on the Iberian Peninsula, the haplogroup composition of the Alto de Reinoso site showed close similarity to the Early and Middle Neolithic of Central Europe. (S11 and S12 Tables). Most interestingly, Middle to Late Neolithic groups in north Spain (NS_MLN) located in the Upper Ebro and Ambrona Valley [76,86] and Alto de Reinoso—though situated in close geographical proximity—showed essential differences at the mitochondrial level (see PCA, Fig 4). The haplogroup composition in north Spain (NS_EN and NS_MLN) appears to have remained relatively unchanged over the course of the Neolithic and a great many haplogroups typical of south-western hunter-gatherer populations (H, U, U5b) still existed in the area. On the other hand, a much greater number of “Neolithic haplogroups” (e.g. J, K, T2, and X) were present in the Early Neolithic north-east Spain, at Alto de Reinoso, and at the nearby Chalcolithic El Mirador site [75] arguing for an influx of Near Eastern Neolithic lineages from north-eastern Spain into the northern Meseta during the Early and Middle Neolithic. The results point to different population-dynamic processes in these regions in the latter stages of the Neolithic period. This stands in contrast to genetic data obtained in Central Europe, where populations seem to have remained rather uniform in their mitochondrial composition during the Early and Middle Neolithic [16,21,86].

### The subsistence and dietary data confirm the impression of a closely related community

Overall, the stable carbon and nitrogen values show that all the members of the community consumed a very similar diet, again confirming the impression of a largely homogenous community. The evidence supporting this assumption is low standard deviations of 0.3 ‰ in δ^13^C and 0.4 ‰ in δ^15^N [130]. The dietary evidence points exclusively to C_3_ plants and a slightly higher consumption of meat and dairy products or increased fertilisation of the farmland compared to contemporaneous cultural groups in Central Europe [131–133]. Increased fertilisation is less likely to occur in the keeping of smaller ruminants due to their usually higher mobility than in cattle and pig farming. Whilst at that time cattle and pigs were predominant in Central Europe, sheep and goats were the preferred sources of food in south-western Europe, where pigs and cattle were rarely part of the diet up to the Late Neolithic [105**–**106].

Furthermore there were no relevant differences in the consumption of food between the sexes or the different age groups within the community. Due to the lack of grave goods, which also underlines the impression of an egalitarian group, any social differentiation between the individuals could be excluded. Though not statistically verified, the females at Alto de Reinoso tended to yield lower nitrogen isotope values than the males (with values more than 0.2 ‰ lower being outside of the range which could be explained by analytical error; Fig 6). Moreover, the data of seven individuals from the top layer were somewhat set apart from the main data cluster due to more negative carbon isotope values (Rein 28, 28/2, 29–33). The lower carbon values could have been the result of different carbon sources in the diet, for example due to the consumption of animals and plants from wooded areas with a distinct canopy [134**–**135].

It is worth noting that all these individuals were from the final phase of use of the megalithic tomb. Besides climate change, which is difficult to identify in short-term variation, other possible reasons for the lower carbon values could have been changes in the subsistence economy such as an increase in legumes whose cultivation requires rather more shaded locations. The data of two individuals (Rein 29 and 32) differ from those of the others by lower carbon and higher nitrogen isotope values. The latter could have been as a result of an increased consumption of animal protein including freshwater fish [136].

Compared to most other sites in Spain, Portugal, and the South of France for which nutritional data are available the nitrogen isotope values at Alto de Reinoso were higher (Fig 6). The only site showing higher δ^15^N value was the Mesolithic site at El Collado near Valencia. However, this site was located on the Spanish Mediterranean coast and the high nitrogen isotope values were probably a result of the consumption of marine fish [137]. The values of Alto de Reinoso are better comparable to late Neolithic data from the Garonne area in the South of France [138]. In this case researchers interpreted the relatively high nitrogen isotope values as the result of a high consumption of animal protein or as a consequence of fertilising practices, and this may also have applied to Alto de Reinoso. Another reason for increased nitrogen values at Alto de Reinoso could have been the aridity in the Meseta compared to other regions on the Iberian Peninsula and in the South of France [111]. The carbon isotope values measured at Alto de Reinoso were similar to those from the Late Neolithic site of Cova de la Pastora [139] in Spain, from Neolithic sites in Portugal (Casa da Moura, Gruta do Caldeirao, Gruta da Feteira, Gruta da Fontainhas, Roche Forte II, Cerca do Zambujal, Melides; [140]) and Late Neolithic sites in the Languedoc area of France [138].

## Conclusion

Methodologically comprehensive and impressively supported by a multitude of results, the study is the first of its kind to provide a more in-depth understanding of the living environment of a burial community such as this. The bioarchaeological data generated have thus allowed us to go beyond the most important individual data such as age and sex and to trace biographical details including familial kinship relations, to reconstruct dietary habits and to highlight patterns of mobility and migration within the region and beyond. Some 50 deceased found their final resting place in the tomb. Based on radiocarbon dates we may state that they covered two or three generations and some of them exhibited close genetic similarities. As shown by the mtDNA profiles the community was composed of a series of independent familial groups whose members generally grew up in the vicinity of Alto de Reinoso. The demographic and genetic composition of the burial community and the reconstruction of its lifestyles and dietary habits, supported by the funerary rites, paint a picture of a close-knit local community whose subsistence was based on farming. With regard to their origins and ancestry the farmers buried at Alto de Reinoso exhibited mainly early farming genetic component, introduced during the Neolithisation of the Iberian Peninsula by immigrants from the Near East, as it is typically seen on the north-eastern part of Spain and in Central Europe. However, a smaller proportion of hunter-gatherer genetic legacy is also reflected in the maternal genetic composition of the population buried at Alto de Reinoso.

The number of bioarchaeological analyses has increased considerably in recent years [141**–**142], although it is still quite rare to find a study that combines a multitude of available scientific methods [20,22**–**23]. The often inadequate social-historical interpretation of such data must, however, be optimised at a meta-level, which will require an even more intensive discourse with archaeological research. For instance the fact that, as today, the societal conditions and social relationships were not necessarily always stable in the past, is often not taken into account. Therefore, we should generally assume that biographies included times of crisis, upheaval and social dislocation and both individuals and communities as a whole were continuously forced to adapt to changing social circumstances. From the point of view of the data generated, however, no particularly unusual individual situations or substantial group-dynamic breaks could be identified that would have impacted on the population of Alto de Reinoso.

## Supporting Information

S1 Fig Example of isolated skulls (Rein 17 and Rein 23) found close to individual Rein 8. (TIF)

S2 Fig Assemblage of a skull (Rein 29) and postcranial bones which appeared like an intentional arrangement. (TIF)

S3 Fig Photo of the individuals buried closely together in the bottom layer. (TIF)

S4 Fig Selection of grave goods from the Neolithic ossuary. Polished axes (1–4); microliths (5–10), blades (15–19) and other flint tools (11–14); bone spatula-idols (20–21); necklace pieces of different raw materials (22–24) and perforated boar tusk (25). Grave goods from the funerary reuse event at the beginning of Bronze Age (SU 2): undecorated pottery bowl (26); bone V-perforated button (27); and archers’ wristguards with incised decoration (28).
(TIF)

S5 Fig Modelling the three ^14^C data measured at the radiocarbon lab MAMS (see S2 Table) using phase analysis of OxCal 4.2 [147–148]. We obtained a period of use of the tomb between 3710–3690 and 3640–3630 cal BC, around 60–80 years.
(DOCX)

S6 Fig Geological map of northern Spain with the localities of Alto de Reinoso and the comparative dataset of Alegria Durantzi. Based on: Geological Map of the Iberian Peninsula, Balearic and Canary Islands", by the Spanish Geomining Technological Institute and Portuguese Geological and Mining Institute, Madrid, 1994, Scale 1:1.000.000. Reproduced with kind permission by the National Geological Institute.
(TIF)

S1 Table Results of osteological, molecular genetic and isotopic analyses for all human individuals sampled from Alto de Reinoso. The numbering of sampled teeth for aDNA and strontium isotope analyses followed FDI pattern (Fédération Dentaire Internationale). Ind. = Individual; Inv. = Inventory Number; Infans I = 0–6; Infans II = 7–12; Juvenile = 13–20; Adult = 21–40; Mature = 41–60; Adult+ = not determinded > 20; m = male; (m) = rather male;? = indetermined; f = female; (f) = rather female. Haplogroup determination is based on Phylotree tree Build 16 (19 Feb 2014).
(XLSX)

S2 Table Radiocarbon data with the 1 sigma and 2 sigma calibrations. Dating of human bone samples from Alto de Reinoso megalithic tomb was carried out in Klaus Tschira Laboratory for Radiometric Dating Methods at Curt Engelhorn Centre Archaeometry gGmbH, Mannheim, Germany.
(XLSX)

S3 Table Minimum number of adults and subadults based on different parts of the skeleton. (DOC)

S4 Table Sex estimation of the Alto de Reinoso individuals based on the left pelvis. (DOCX)

S5 Table Sex determination of the Alto de Reinoso individuals based on the crania of 21 adults. (DOCX)

S6 Table Demographic information about the Alto de Reinoso community based on age and sex estimations of the crania and mandibles. (DOCX)

S7 Table Results of the GenoCoRe 22. SNP profiles are presented in comparison to the revised Cambridge Reference Sequence (rCRS; [149]). SNPs can either be detected in forward (L-strand) or in reverse direction (H-strand). Underlined SNPs in the headline were detected in reverse direction. Derived SNPs are colored in dark gray, derived SNPs with a peak height fewer than 50 in light gray. Ancestral SNPs that had a peak height lower than 50 are marked in bold. A dash indicates an allelic dropout.
(XLSX)

S8 Table Results of the H-Plex [70]. SNP profiles are presented in comparison to the revised Cambridge Reference Sequence (rCRS; [149]). Derived SNPs are colored in dark gray, derived SNPs with a peak height fewer than 50 in light gray. Ancestral SNPs that had a peak height lower than 50 are marked in bold. A dash indicates an allelic dropout.
(XLSX)

S9 Table HVR I and HVR II primers sequences. HVR I primers are named after the last 5ˈ base of the primer, HVR II primers are named after the first base or last base respectively of the amplified DNA fragment.
(XLSX)

S10 Table Strontium and carbon and nitrogen isotopes analyses on faunal samples. Results of the strontium isotope analysis in the faunal remains used as comparative data for the local range at Alto de Reinoso and results of the carbon and nitrogen isotope analysis of the faunal remains from Alto de Reinoso.
(XLSX)

S11 Table Summary of prehistoric comparative mtDNA data. The palaeogenetic information over various archaeological sites was classified according to archaeological, chronological information and/or geographical distribution.
(XLSX)

S12 Table Relative mtDNA haplogroup frequencies used for the principal component analysis. Culture information and references to comparative data are presented in S10 Table.
(XLSX)

S1 Text Report on all methods and protocols applied in this study. (DOC)
