# Supplementary figures and images for: A Community in Life and Death: The Late Neolithic Megalithic Tomb at Alto de Reinoso (Burgos, Spain)
Source: PLoS One. 2016 Jan 20;11(1):e0146176. doi: 10.1371/journal.pone.0146176 (PMC4720281; doi:10.1371/journal.pone.0146176)

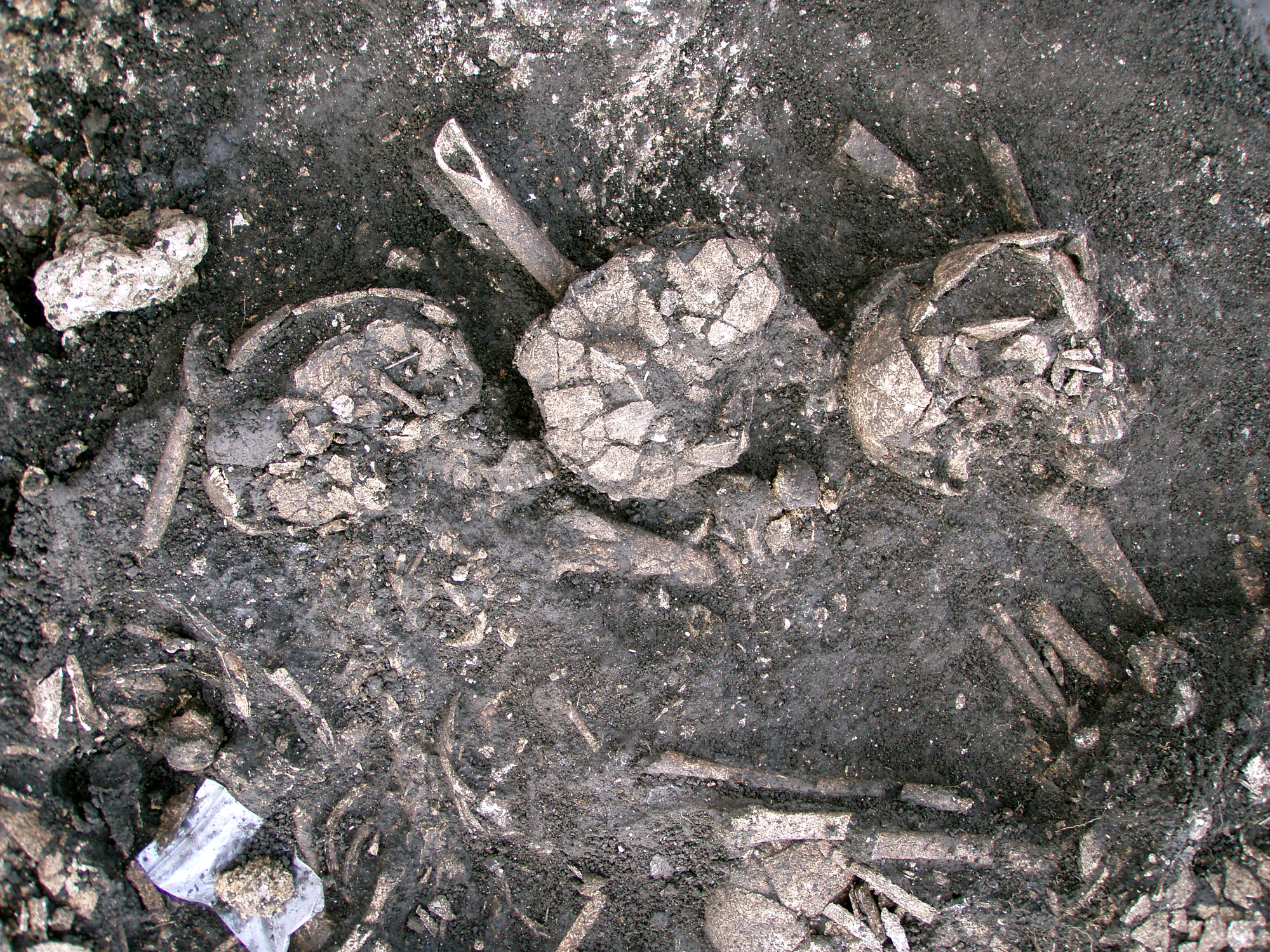

Supplement: S1 Fig — (TIF) [file pone.0146176.s001.tif]

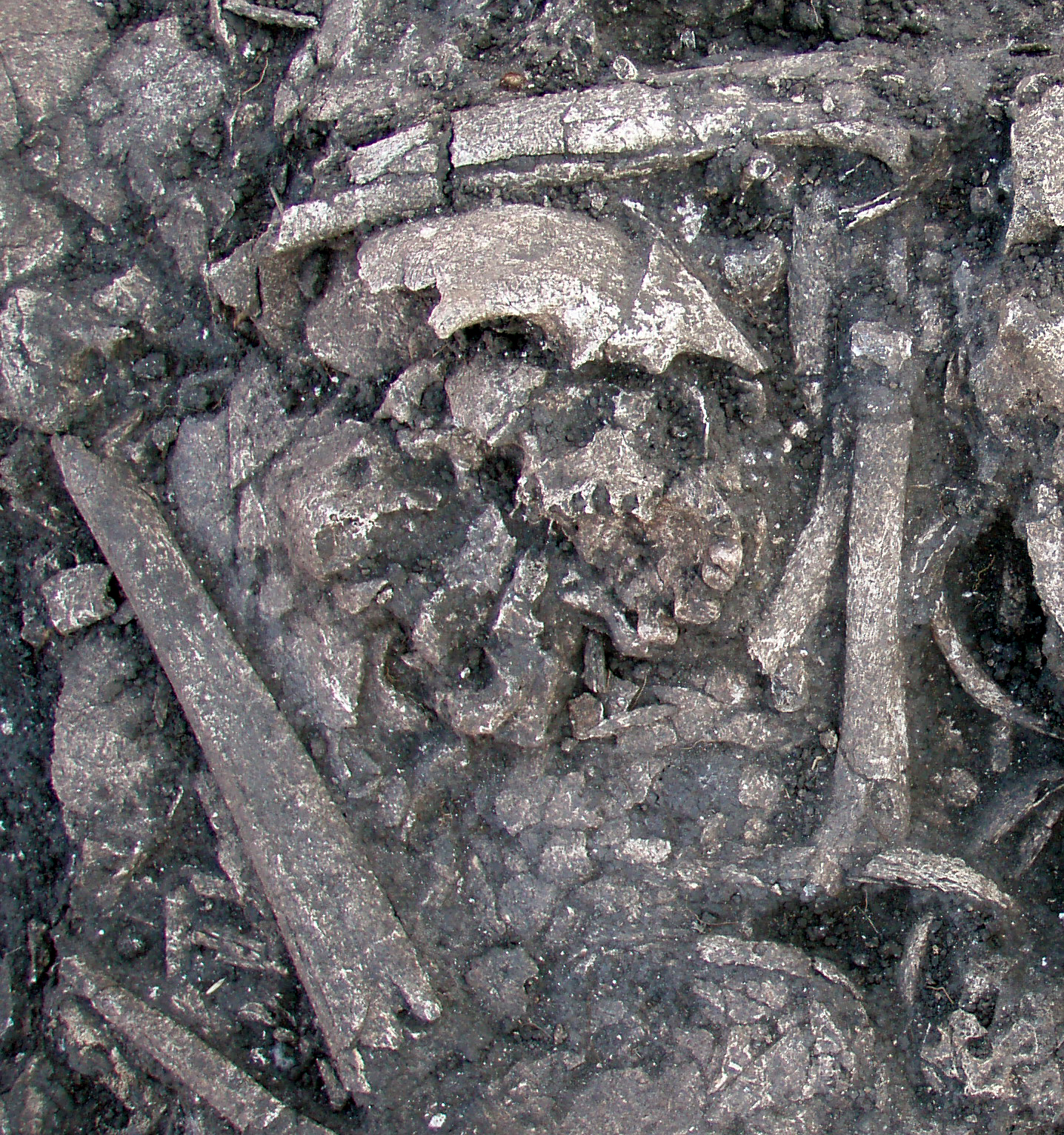

Supplement: S2 Fig — (TIF) [file pone.0146176.s002.tif]

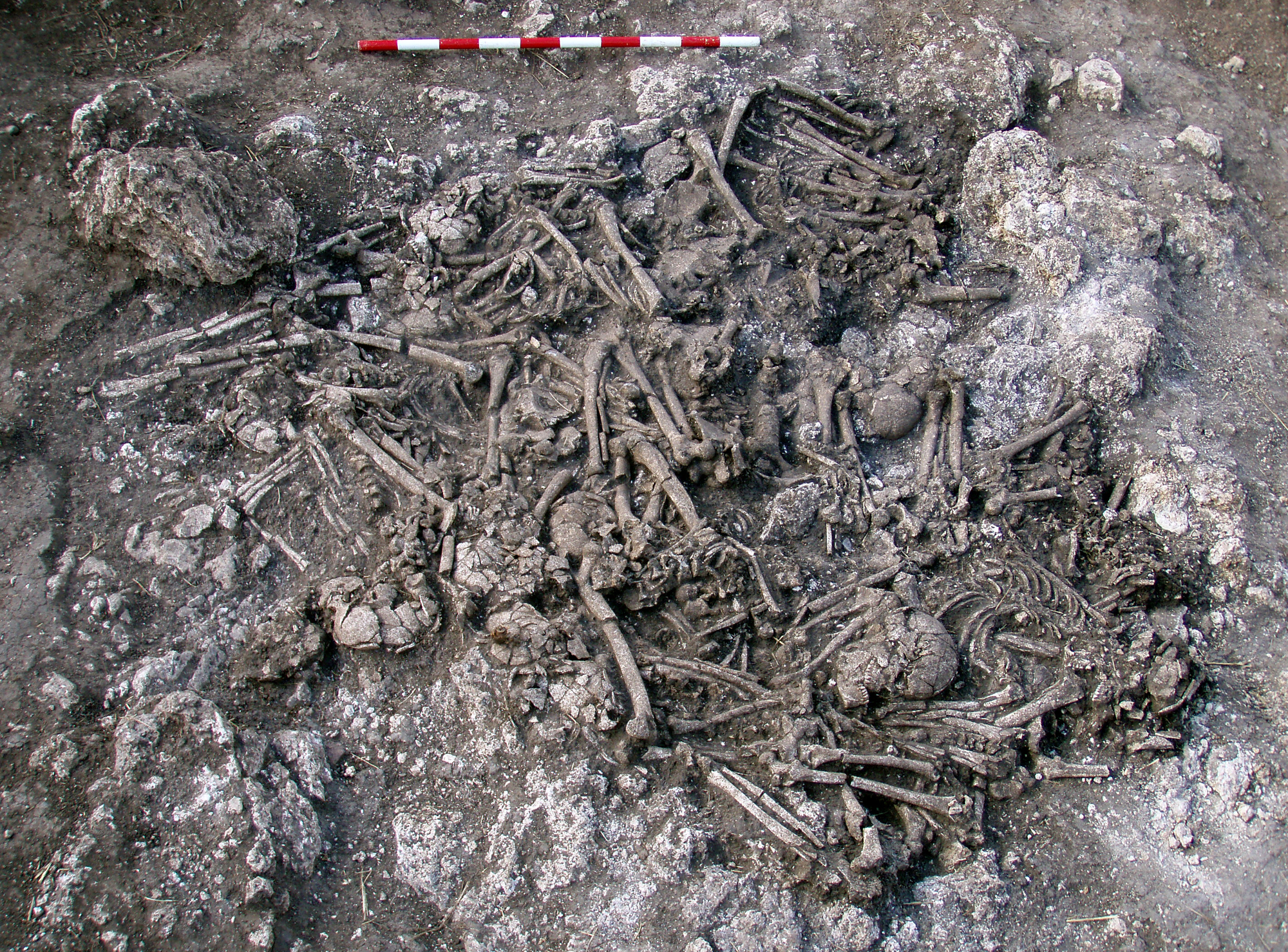

Supplement: S3 Fig — (TIF) [file pone.0146176.s003.tif]

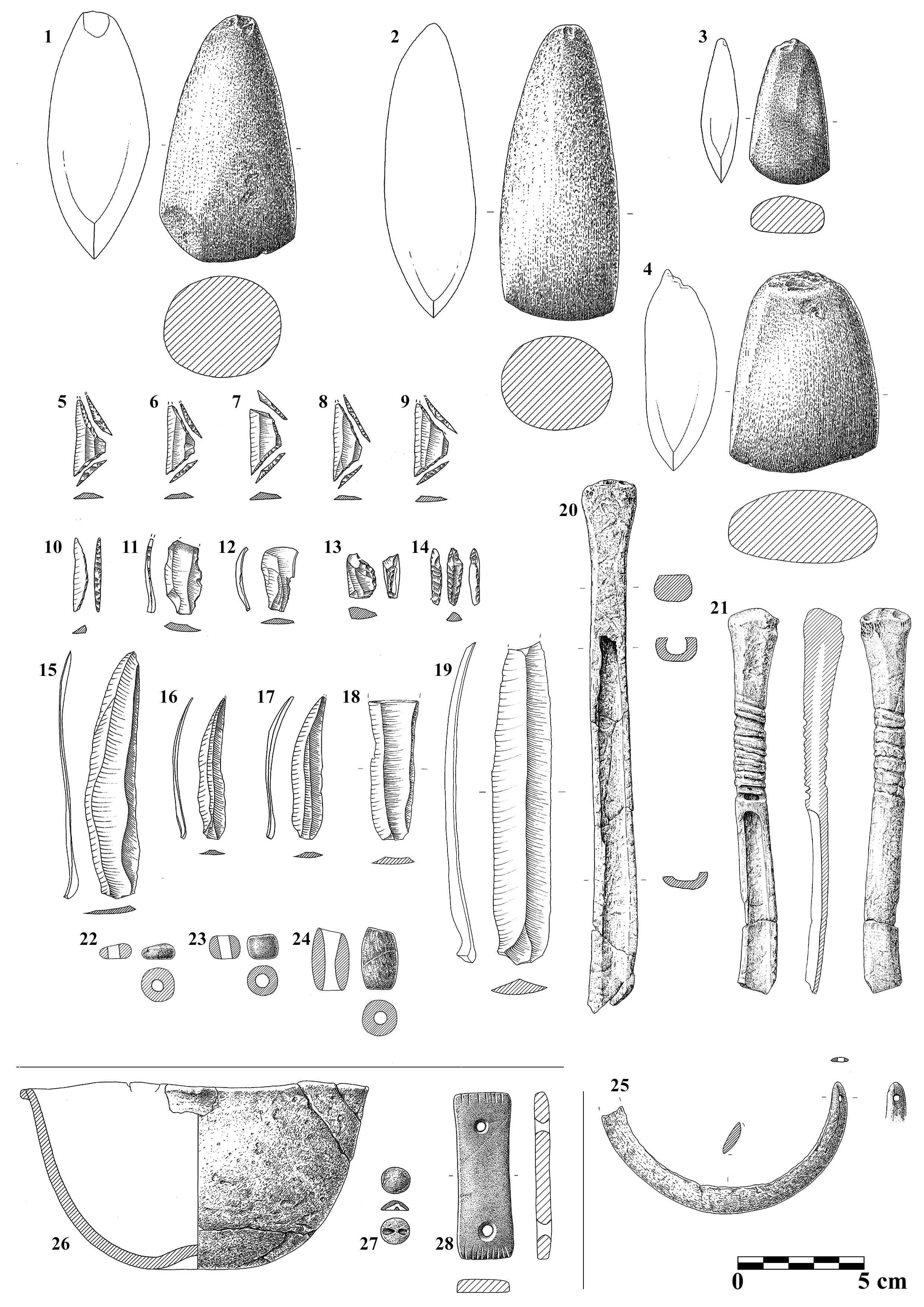

Supplement: S4 Fig — Polished axes (1–4); microliths (5–10), blades (15–19) and other flint tools (11–14); bone spatula-idols (20–21); necklace pieces of different raw materials (22–24) and perforated boar tusk (25). Grave goods from the funerary reuse event at the beginning of Bronze Age (SU 2): undecorated pottery bowl (26); bone V-perforated button (27); and archers’ wristguards with incised decoration (28). (TIF) [file pone.0146176.s004.tif]

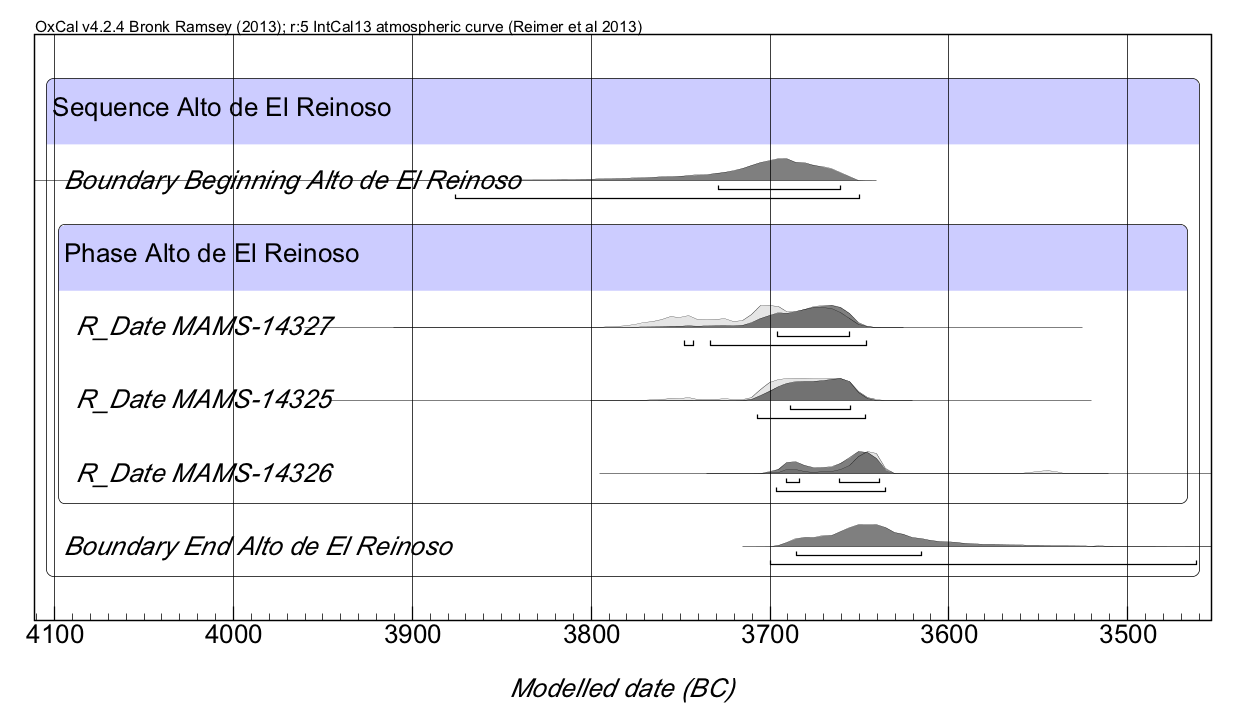

Supplement: S5 Fig — We obtained a period of use of the tomb between 3710–3690 and 3640–3630 cal BC, around 60–80 years. (DOCX) [file pone.0146176.s005.docx]

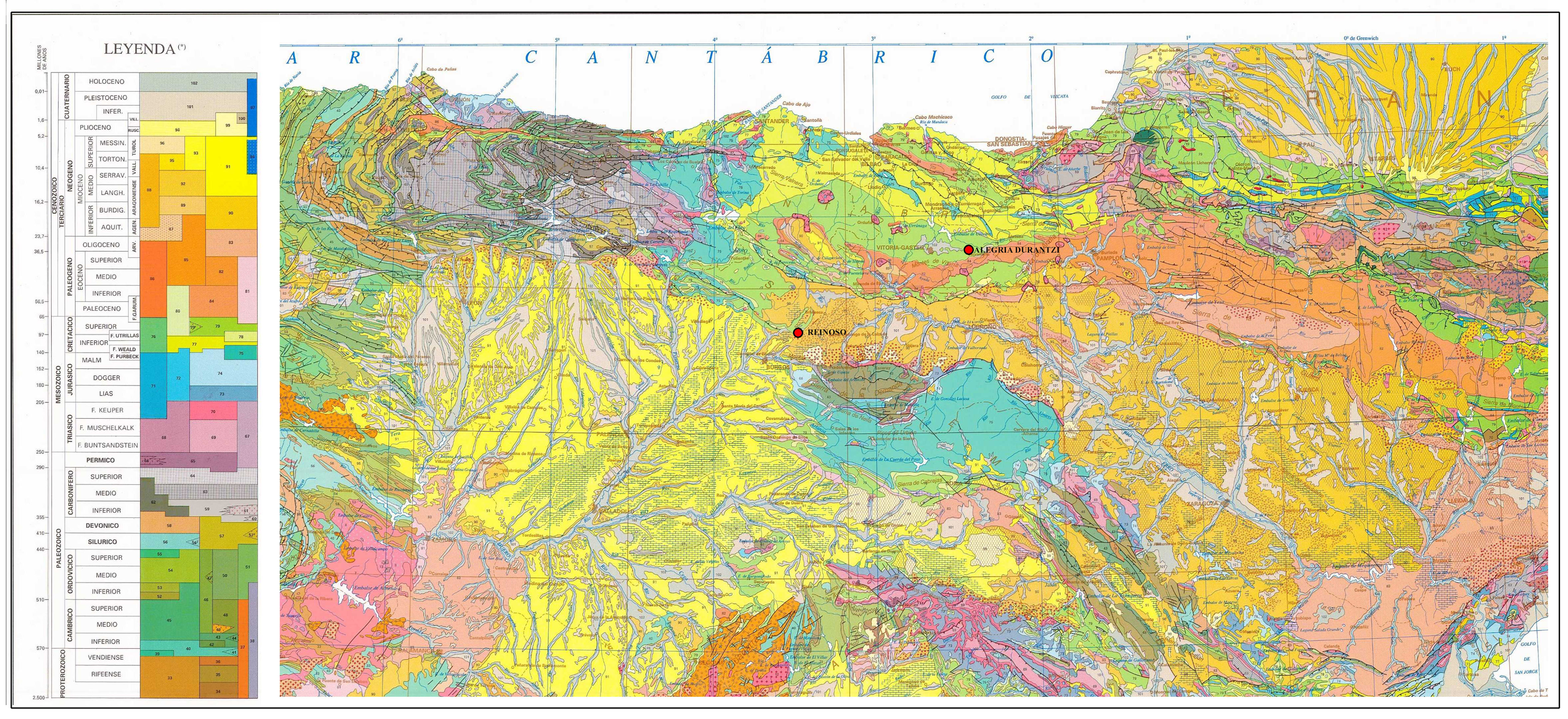

Supplement: S6 Fig — Based on: Geological Map of the Iberian Peninsula, Balearic and Canary Islands", by the Spanish Geomining Technological Institute and Portuguese Geological and Mining Institute, Madrid, 1994, Scale 1:1.000.000. Reproduced with kind permission by the National Geological Institute. (TIF) [file pone.0146176.s006.tif]
