# Supplementary material for: A Community in Life and Death: The Late Neolithic Megalithic Tomb at Alto de Reinoso (Burgos, Spain)
Source: PLoS One. 2016 Jan 20;11(1):e0146176. doi: 10.1371/journal.pone.0146176 (PMC4720281; doi:10.1371/journal.pone.0146176)
Supplement: S3 Table — (DOC) [file pone.0146176.s009.doc]

**S3 Table. Minimum number of adults and subadults based on different parts of the skeletons.**

| Skeletal part | Adult (> 20 y) | Subadult (< 20 y) | Total |
| --- | --- | --- | --- |
| crania | 21 | **17** | 38 |
| maxilla, right | 14 | 14 | 28 |
| maxilla, left | 19 | 12 | 31 |
| mandible, right | 22 | 16 | 38 |
| mandible, left | 21 | 11 | 32 |
| pelvis, right | 22 | 9 | 31 |
| pelvis, left | 26 | 7 | 33 |
| femur, right | **30** | 9 | 39 |
| femur, left | 26 | 11 | 37 |
