# Supplementary material for: A Community in Life and Death: The Late Neolithic Megalithic Tomb at Alto de Reinoso (Burgos, Spain)
Source: PLoS One. 2016 Jan 20;11(1):e0146176. doi: 10.1371/journal.pone.0146176 (PMC4720281; doi:10.1371/journal.pone.0146176)
Supplement: S4 Table — (DOCX) [file pone.0146176.s010.docx]

**S4 Table. Sex estimation of the Alto de Reinoso individuals based on the left pelvis.**

|  | *Female* | *Female?* | *Male* | *Male?* | *Indet.* | *Total* |
| --- | --- | --- | --- | --- | --- | --- |
| Count | 1 | 11 | 4 | 7 | 4 | 27 |
| **%** | 3.7 | 40.7 | 14.8 | 25.9 | 14.8 | 100 |
