# Supplementary material for: A Community in Life and Death: The Late Neolithic Megalithic Tomb at Alto de Reinoso (Burgos, Spain)
Source: PLoS One. 2016 Jan 20;11(1):e0146176. doi: 10.1371/journal.pone.0146176 (PMC4720281; doi:10.1371/journal.pone.0146176)
Supplement: S5 Table — (DOCX) [file pone.0146176.s011.docx]

**S5 Table. Sex determination of the Alto de Reinoso individuals based on the crania of 21 adults.**

|  | Female | Female? | Male | Male? | Indet. | Total |
| --- | --- | --- | --- | --- | --- | --- |
| Count | 0 | 5 | 3 | 6 | 7 | 21 |
| % | 0.0 | 23.8 | 14.3 | 28.6 | 33.3 | 100 |
