# Supplementary material for: A Community in Life and Death: The Late Neolithic Megalithic Tomb at Alto de Reinoso (Burgos, Spain)
Source: PLoS One. 2016 Jan 20;11(1):e0146176. doi: 10.1371/journal.pone.0146176 (PMC4720281; doi:10.1371/journal.pone.0146176)
Supplement: S6 Table — (DOCX) [file pone.0146176.s012.docx]

**S4 Table. Demographic information about the Alto de Reinoso community based on age and sex estimations of the crania and mandibles.**

|  | *Female* | *Female?* | *Male* | *Male?* | *Indet.* | *Total* | *%* |
| --- | --- | --- | --- | --- | --- | --- | --- |
| Infans I |  |  |  |  | 4 | 4 | 10.5 |
| Infans II |  |  |  |  | 5 | 5 | 13.2 |
| Juvenile |  |  | 2 |  | 6 | 8 | 21.0 |
| **Subadults** |  |  | 2 |  | 15 | 17 | 44.7 |
| Adult |  | 2 | 2 | 4 | 4 | 12 | 31.5 |
| Mature |  | 2 | 1 |  | 1 | 4 | 10.5 |
| Senile |  |  |  |  |  |  |  |
| Adult+ |  | 1 |  | 2 | 2 | 5 | 13.2 |
| **Adults** |  | 5 | 3 | 6 | 7 | 21 | 55.3 |
| **Total** |  | 5 | 5 | 6 | 22 | 38 | 100.0 |

Infans I=0–6; Infans II=7–12; Juvenile=13–20; Adult=21–40; Mature=41–60; Senile≥61; Adult+≥20; Indet.= indetermined
