# Supplementary material for: A Community in Life and Death: The Late Neolithic Megalithic Tomb at Alto de Reinoso (Burgos, Spain)
Source: PLoS One. 2016 Jan 20;11(1):e0146176. doi: 10.1371/journal.pone.0146176 (PMC4720281; doi:10.1371/journal.pone.0146176)
Supplement: S1 Text — (DOC) [file pone.0146176.s019.doc]

S1 Text

Analytical methods

*Archaeology*

The extraordinary density of this complex ossuary demanded a specific methodology of recording the archaeological evidence [1]. A total station was used to locate every single bone once it was excavated (Leica TS02). Measurements at the two edges of the long bones were recorded, first that closer to the head of the individual. Skulls, scapulae or pelvis were documented by several characteristic points around their contour. Finally, thousands of registers were taken which exactly located every bone inside the mass of this complex ossuary, which were later processed with ArcGis software (ArcGis 10.0). Every anatomical connection was documented photographed and the bones separated and a record number given. The same was done with the ritual arrangements or assemblages of bones documented. All this permits to exactly locate the position and orientation of every bone inside the ossuary. The documentation was completed with the exhaustive photographic record and the archaeological drawing of several successive plans. The specimen numbers of all samples analysed in our study are listed in the supplementary information S1 Text (Table S1). The complete repository information and all details of permits of all aspects of this study are given in a declaration attached to this submission.

*Osteology*

The determination of the minimum number of individuals (MNI) is based on the most often preserved skeletal elements [2]. To estimate age at death of the individuals the following features were assessed: tooth development and eruption [3], tooth wear [4,5], ectocranial suture closure [6], ossification of the vertebral end plates [7], length of long bones [8,9], epiphyseal closure of long bones, and presence of degenerative joint alterations [10]. The morphologic sex determination was based on the assessment of sex specific traits on the pelvis and skull [9-10]. Diameter and circumference of long bones were measured and analyzed regarding their sex specific variation where preservation allowed it [11]. Sex analysis of subadults was not possible. The estimation of body height was based on studies of Pearson [12] for non-accelerated populations as well as on Breitinger [13] and Bach [14]. The diagnosis of pathologies, traumata, and stress markers was only possible in some cases due to poor preservation [15-20]). Taphonomic processes and anthropogenic disturbances of the skeletal remains that were involved in creating the complexity of the burial findings are discussed thoroughly [21].

*Molecular genetics*

Samples from Reinoso were taken with face masks and gloves prior to any anthropological analyses except for the samples of Rein 23 and Rein 30 which were taken during the osteological investigations. For each putative individual two to three teeth were sampled except for the individuals Rein 6, Rein 7, and Rein 9, for which only bones were available. For the latter two different long bones were sampled (samples A and B). Sample C of individual Rein 6 was used from a different part of the same bone as the A sample.

In this study we generated mitochondrial profiles of 27 individuals by analyzing the hypervariable regions I and II (HVR I, HVR II) and coding region single nucleotide polymorphisms (SNPs) of the mitochondrial genome (Table S7, S8, S11). Protocols for sample preparations, extraction, amplification setups, and contamination control were applied as described previously [22-25]. After decontamination and cleaning of the samples by UV-C irradiation and a sandblasting tool, we ground them into fine powder, and extracted DNA using the phenol-chloroform method [24]. The PCR set-up and thermal conditions are reported in our previous studies [24-25], new primer pairs are listed in Table S11. The PCR clean up, sequencing and cloning were also performed following the previously described protocols [24-25].

As a minimum all results were reproduced either by three PCR of two extractions with sporadic cloning or by two PCR of two extractions with complete cloning of all PCR products. For certain individuals of the haplogroups (hg) K und U with matching haplotypes in HVR I, the HVR II was amplified via 4 overlapping fragments from each extract. Additionally, 22 coding region SNPs defining the major Eurasian haplogroups were amplified in a multiplex approach for at least two samples of all individuals (GenoCoRe22, [23]). For individual Rein 28 a similar assay was conducted but targeting 17 coding regions SNPs of subgroups of haplogroup H frequent in extant Europe [26]. Haplogroup assignments were made based on the results of HVR I and II sequences, coding region SNPs, according to the phylogeny of PhyloTree.org (mtDNA tree Build 16 [19 Feb 2014], [27]) were considered. For one individual (Rein 10) the haplogroup (hg) was successfully reproduced in two extractions with the GenoCoRe22, the HVR I, however, showed two different reproducible lineages; one lineage of hg K and one of hg H. Therefore, the GenoCoRe22 results were used for statistical analyses, the HVR I profile, however, was not regarded as authenticated. The HVS-I mtDNA consensus sequences were uploaded to GenBank ([www.ncbi.nlm.nih.gov/genbank](http://www.ncbi.nlm.nih.gov/genbank)) under the accession numbers KT868907-KT868932.

The HVR II, which was mainly analyzed to differentiate individuals with identical HVR I sequences, proofed to be difficult to amplify for some individuals (Rein 2, 12, 14, and 24). For Rein 12 and 14 it was not possible to reproduce the last fragment of the HVR II by a second sample, however, all polymorphisms are reproduced by two different samples. For Rein 24 and 2 some SNPs were not reproduced by a second sample and therefore denoted in brackets in supplement (Table S1). However, because a certain degree of variability on the HVR II was recognizable and important for the discussion of putative maternal kinship relations, these incomplete HVR II data were included in the discussion.

Authentication of mtDNA results was established according to a number of parameters as has been discussed in numerous papers on ancient DNA [22,24]. Only individuals whose haplotypes could be reproduced over the whole HVR I by several PCRs of at least two extractions were set as authentic. In all cases, the mitochondrial HVR I and II sequences supported the results of the GenoCoRe22 multiplex. Authenticity was only considered established when identical results were obtained from at least two independently extracted samples, while the detection of identical contaminating lineages in two different extractions of one individual led to the exclusion from the sample set.

Mitochondrial profiles of genetic investigators and people involved in sampling and excavation were sequenced and compared to the aDNA results in order to monitor potential contamination but this did not occur. Only the haplotype of one genetic investigator who was involved mainly in sample preparation coincided with the haplotype of Rein 28. No signs of contaminations were found in any of the other samples he handled at the same time. Above all, the shared CRS-lineage is a very common haplotype, both nowadays and in prehistory, arguing for a coincidental haplotype match with the ancient sample. For all other analysed samples no haplotype match to any archaeologist or genetic researcher of the study could be found. The overall contamination rate detected in PCR, extraction and milling blanks was 2.15% during the course of the three years long laboratory work, in which several sets of samples were processed parallel. The positive blanks were sequenced and compared to the samples.

For statistical analyses data were pooled according to chronology and geographical location into a hunter-gatherer group with Paleolithic to Mesolithic data from Spain and Portugal (HGSW) [28-32], three Early Neolithic groups from Northeast Spain (NES_EN) [33-35], the northern Spain Upper Ebro Valley (NS_EN) [30,33] and Central Portugal (CP_EN) [28-29], dating from ca. 5500 to 4500 cal BC, and three later Neolithic groups, from Northeast Spain (NES_MLN) [36,33] and the north Spanish Upper Ebro and Ambrona Valley (NS_MLN) [30,35], dating between ca. 4200 and 3000 cal BC and the Chalcolithic El Mirador site [37] and a Late Neolithic-Chalcolithic Portugal group (CP_LN_CH) [28-29]). Neolithic data from 13 other European groups – hunter-gatherers (HGC) [38-43], Starčevo culture (STA) and Linear Pottery culture in Transdanubia (LBKT) [39], Linear Pottery culture in Central Europe (LBK), Rössen (RSC), Schöningen (SCG), Salzmünde (SMC), Baalberge (BAC), Bernburg (BEC) in Central Europe [44,23-24,45] and south French Treilles culture (TRE) [34] – covering the complete time span of the Iberian comparative samples were included into the analyses. To characterize and compare each group based on their haplogroup frequencies, categorical PCA was carried out with 13 prehistoric groups of Central Europe and the Iberian Peninsula (Table S10a), using the prcomp function for categorical PCA, implemented in the R 3.0.2 [46]. Haplogroups were divided into the following 21 (sub-) groups, which were observed in the ancient mtDNA data: H, H5, HV, I, J, K, R, N*, N1a, T1, T2, U, U2, U3, U4, U5a, U5b, U8, V, W, X (Table S10b, Figure 4a).

Adjoining to certain PCAs, we performed hierarchical clustering analyses with Ward type algorithm and Euclidean similarity measurement method [47]. All PCs (all genetic variations) were used for the clustering. The Ward clustering results were visualized in R as a dendrogram by using the hclust function in R.2.13.1. Cluster significance was given as AU (Approximately Unbiased) p-value in %, which is computed by multi-scale bootstrap resampling with 10,000 replicates (Figure 4b).

*Strontium isotope analysis*

Sampling and pretreatment of enamel and bones for strontium isotope analysis were conducted at the Institute for Anthropology at Mainz University following Knipper et al. [48]. Enamel chips were either separated during sample preparation for aDNA analyses or cut from complete teeth using a diamond coated dental cutting disc. Thorough removal of all surfaces and dentin residues was essential prior to powdering the samples in an agate mortar. In several cases the powder was drilled directly from the teeth or bones after their cleaning with de-ionised water in an ultrasonic bath and removal of surfaces and discolored portions.

The pretreatment of the sample powders involved soaking in de-ionised H_2_O and 0.1 M acetic acid buffered with a Li-acetate (pH 4.5) for 10 min in an ultrasonic bath and three rinses with de-ionized water. Samples were dried overnight (50°C) and ashed for three hours at 850°C. Strontium separation with Eichrom Sr-Spec resin was carried out in the clean laboratory facilities at the Curt Engelhorn Centre Archaeometry gGmbH in Mannheim following the procedures described in Knipper et al. [148]. Strontium concentrations were determined by a Quadrupole-Inductively Coupled Plasma-Mass Spectrometry (Q-ICP-MS) and the ^87^Sr/^86^Sr ratios by a Multi Collector-ICP-MS (VG Axiom). Raw data were corrected according to the exponential mass fractionation law to ^88^Sr/^86^Sr = 8.375209. Blank values were lower than 10 pg Sr during the whole clean lab procedure. The Eimer & Amend (E & A) standards run along with the human and modern comparative samples yielded ^87^Sr/^86^Sr ratios of 0.70803 ± 0.00007 (2 SD; n = 42). The long-term inter-laboratory average of this standard is 0.708027 ± 0.000035 (1σ) [49].

*Carbon and nitrogen isotope analysis*

Samples for carbon and nitrogen isotope analyses comprised skull fragments and in one case a rib of 25 individuals which were sampled for aDNA and from 4 additional individuals. Additionally six faunal bones were sampled for comparison. Collagen extraction followed Longin [50] with some modifications [51-52]. The bone surfaces were manually removed and cleaned with dental cutting and milling equipment. Bone samples were demineralized in 10 ml of 0.5 N HCL for about 14 days. After neutralization with dionized water the samples were treated with 0.1 M NaOH for 24 h to dissolve humic acids and rinsed again to neutrality. Gelatinization was conducted at 70°C for 48 h. Collagen was then filtered with Ezee-Filter™ separators (Elkay) and concentrated using Amicon© ultrafilters (Millipore; cutoff, <30 kDa). The purified collagen was frozen and lyophilized for 48 h. One to two milligrams of dried collagen was weighed into tin capsules. The Carbon and nitrogen contents were determined by an elemental analyzer (vario EL III, Elementar Analytical Systems) and the isotope ratios were by an IsoPrime High Performance isotope ratio mass spectrometer (IRMS; VG Instruments). All samples were run in duplicates. The stable isotope ratios are quoted in δ-notation in per mille relative to VPDB for carbon and AIR for nitrogen. Normalization of the data was performed using two-point calibrations based on USGS 40 and IAEA N2 for nitrogen and CH6 and CH7 for carbon [53]. Measurement errors were smaller than ± 0.2 ‰ for nitrogen and ± 0.1 ‰ for carbon (1 SD). For statistical analysis IBM SPSS Statistics software Version 19 for Windows (SPSS Inc.) was used.

References

1. Harris EC. Principles of archaeological stratigraphy. London, New York: Academic Press; 1979.
2. Marshall F, Pilgram T. NISP vs. MNI in quantification of body-part representation. Am Antiq. 1993;58: 261‒269.
3. Ubelaker DH. Human skeletal remains: excavation, analysis, interpretation. 2nd ed. Washington, DC: Taraxacum; 1989.
4. Miles AEW. The dentition in the assessment of individual age in skeletal material. In: Brothwell DR, editor. Dental Anthropology. Symposia of the Society for the Study of Human Biology 5. Oxford: Symposium Publications Division, Pergamon Press; 1963. pp. 191−209.
5. Lovejoy CO. Dental wear in the Libben population: its functional pattern and role in the determination of adult skeletal age at death. Am J Phys Anthropol. 1985;68: 47−56.
6. Meindl RS, Lovejoy CO. Ectocranial suture closure: a revised method for the determination of skeletal age at death based on the lateral-anterior sutures. Am J Phys Anthropol. 1985;68: 57−66.
7. Albert AM, Maples WR. Stages of epiphyseal union for thoracal and lumbar vertebral centra as a method of age determination for teenage and young adult skeletons. J Forensic Sci. 1995;40: 623−33.
8. Stloukal M, Hanáková H. Die Länge der Längsknochen altslawischer Bevölkerungen – Unter besonderer Berücksichtigung von Wachstumsfragen. Homo. 1978;29: 53−69.
9. Buikstra JE, Ubelaker DH. Standards for data collection from human skeletal remains. Fayetteville: Arkansas Archaeological Survey Series 44, 1994.
10. Ferembach D, Schwidetzky I, Stloukal M. Recommendations for age and sex diagnoses of skeletons. J Hum Evol. 1980;9: 517–549.
11. Mays S, Cox M. Sex determination in skeletal remains. In: Cox M, Mays S, editors. Human osteology in archaeology and forensic science. London: Greenwich Medical Media; 2002. pp. 117‒130.
12. Pearson K. Mathematical contributions to the theory of evolution. V. On the reconstruction of the stature of prehistoric races. Philos Trans R Soc Lond A. 1899;192: 169‒245.
13. Breitinger E. Zur Berechnung der Körperhöhe aus den langen Gliedmaßenknochen. Anthropol Anz. 1938;14: 249‒274.
14. Bach H. Zur Berechnung der Körperhöhe aus den langen Gliedmaßenknochen weiblicher Skelette. Anthropol Anz. 1965;29: 12‒21.
15. Aufderheide AC, Rodríguez-Martín C. The cambridge encyclopedia of human paleopathology. Cambridge: Cambridge University Press; 1998.
16. Schultz M. Paläopathologische Diagnostik. In: Knußmann R, editor. Anthropologie. Handbuch der vergleichenden Biologie des Menschen Vol I, 1. Stuttgart: Fischer; 1988. pp. 480‒496.
17. Schultz M, Carli-Thiele P, Schmidt-Schultz TH, Kierdorf U, Kierdorf H, Teegen W-R, et al. Enamel hypoplasias in archaeological skeletal remains. In: Alt KW, Rösing FW, Teschler-Nicola M, editors. Dental anthropology. Fundamentals, limits, and prospects. Wien, New York: Springer; 1998. pp. 293‒311.
18. Caselitz P. Caries - ancient plague of humankind. In: Alt KW, Rösing FW, Teschler-Nicola M, editors. Dental anthropology. Fundamentals, limits, and prospects. Wien, New York: Springer; 1998. pp. 203‒226.
19. Ortner DJ. Identification of pathological conditions in human skeletal remains. 2nd ed. San Diego: Academic Press; 2003.
20. Witzel C. Morphological analysis of enamel hypoplasias as markers of systemic stress – a contribution to the patho-biography of humans and animals. Ph.D Thesis, The University of Hildesheim. 2009.
21. Haglund WD, Sorg MH. Advances in forensic taphonomy: methods, theory, and archaeological perspectives. Boca Raton: CRC Press; 2002.
22. Haak W, Brandt G, de Jong HN, Meyer C, Ganslmeier R, Heyd V, et al. Ancient DNA, strontium isotopes, and osteological analyses shed light on social and kinship organization of the Later Stone Age. Proc Natl Acad Sci U S A. 2008;105: 18226‒18231.
23. Haak W, Balanovsky O, Sanchez JJ, Koshel S, Zaporozhchenko V, Adler CJ, et al. Ancient DNA from European early Neolithic farmers reveals their Near Eastern affinities. PLoS Biol. 2010;8: doi:org/10.1371/journal.pbio.1000536
24. Brandt G, Haak W, Adler C J, Roth C, Szécsényi-Nagy A, Karimnia S, et al. Ancient DNA reveals key stages in the formation of Central European mitochondrial genetic diversity. Science. 2013;342: 257‒261.
25. Knipper C, Meyer C, Jacobi F, Roth C, Fecher M, Stephan E, et al. Social differentiation and land use at an Early Iron Age "princely seat": bioarchaeological investigations at the Glauberg (Germany). J Archaeol Sci. 2014;41: 818‒835.
26. Martínez-Cruz B, Harmant C, Platt DE, Haak W, Manry J, Ramos-Luis E, et al. Evidence of pre-Roman tribal genetic structure in Basques from uniparentally inherited markers. Mol Biol Evol. 2012;29: 2211–2222.
27. van Oven M, Kayser M. Updated comprehensive phylogenetic tree of global human mitochondrial DNA variation. Hum Mutat. 2009;30: E386-E394. http://www.phylotree.org. doi: 10.1002/humu.20921
28. Chandler HC. Using ancient DNA to link culture and biology in human populations. Ph.D. Thesis. The University of Oxford. 2003.
29. Chandler H, Sykes B, Zilhão J. Using ancient DNA to examine genetic continuity at the Mesolithic-Neolithic transition in Portugal. In: Arias P, Ontañón R, García-Moncó C, editors. Actas del III Congreso del Neolítico en la Península Ibérica, Santander, Monografías del Instituto internacional de Investigaciones Prehistóricas de Cantabria 1. Santander: Servicio de Publicaciones, Universidad de Cantabria; 2005. pp. 781–786.
30. Hervella M, Izagirre N, Alonso S, Fregel R, Alonso A, Cabrera VM, et al. Ancient DNA from hunter-gatherer and farmer groups from Northern Spain supports a random dispersion model for the Neolithic expansion into Europe. PloS One. 2012;7: doi:org/10.1371/journal.pone.0034417
31. Sánchez-Quinto F, Schroeder H, Ramirez O, Avila-Arcos MC, Pybus M, Olalde I, et al. Genomic affinities of two 7,000-year-old Iberian hunter-gatherers. Current Biology .2012;22: 1494–99.
32. de-la-Rua C, Izagirre N, Alonso S, Hervella M. Ancient DNA in the Cantabrian fringe populations: a mtDNA study from Prehistory to Late Antiquity. Quat Int. 2015;364: 306–311.
33. Gamba C, Fernández E, Tirado M, Deguilloux MF, Pemonge MH, Utrilla P, et al. Ancient DNA from an Early Neolithic Iberian population supports a pioneer colonization by first farmers. Mol Ecol. 2011;21: 45–56.
34. Lacan M, Keyser C, Ricaut F-X, Brucato N, Duranthon F, Guilaine J, et al. Ancient DNA reveals male diffusion through the Neolithic Mediterranean route. Proc Natl Acad Sci U S A. 2011a;108: 9788–9791.
35. Haak W, Lazaridis I, Patterson N, Rohland N, Mallick S, Llamas B, et al. Massive migration from the steppe was a source for Indo-European languages in Europe. Nature. 2015;doi:10.1038/nature14317
36. Sampietro ML, Lao O, Caramelli D, Lari M, Pou R, Martí M, et al. Palaeogenetic evidence supports a dual model of Neolithic spreading into Europe. Proc R Soc B. 2007;274: 2161–2167.
37. Gómez-Sánchez D, Olalde I, Pierini F, Matas-Lalueza L, Gigli E, Lari M, et al. Mitochondrial DNA from El Mirador cave (Atapuerca, Spain) reveals the heterogeneity of Chalcolithic populations. PLoS One. 2014;9: doi:10.1371/journal.pone.0105105
38. Lazaridis I, Patterson N, Mittnik A, Renaud G, Mallick S, Kirsanow K, et al. Ancient human genomes suggest three ancestral populations for present-day Europeans. Nature. 2014;513: 409–413.
39. Szécsényi-Nagy A, Brandt G, Haak W, Keerl V, Jakucs J, Möller-Rieker S, et al. Tracing the genetic origin of Europe’s first farmers reveals insights into their social organization. Proc R Soc B. 2015;282: doi: 10.1098/rspb.2015.0339
40. Bramanti B, Thomas MG, Haak W, Unterländer M, Jores P, Tambets K,et al. Genetic discontinuity between local hunter-gatherers and central Europe’s first farmers. Science. 2009;326: 137–140.
41. Bollongino R, Nehlich O, Richards MP, Orschiedt J, Thomas MG, Sell C, et al. 2000 years of parallel societies in Stone Age Central Europe. Science. 2013;342: 479–481.
42. Fu Q, Mittnik A, Johnson PLF, Bos K, Lari M, Bollongino R, et al. A revised timescale for human evolution based on ancient mitochondrial genomes. Current Biology. 2013;23: 553–559.
43. Gamba C, Jones ER, Teasdale MD, McLaughlin RL, Gonzalez-Fortes G, Mattiangeli V, et al. Genome flux and stasis in a five millennium transect of European prehistory. Nat Commun. 2014;5: doi:10.1038/ncomms6257
44. Haak W, Forster P, Bramanti B, Matsumura S, Brandt G, Tänzer M, et al. Ancient DNA from the first European farmers in 7500-year-old Neolithic sites. Science. 2005;310: 1016–1018.
45. Lee EJ, Krause-Kyora B, Rinne C, Schütt R, Harder M, Müller J, et al. Ancient DNA insights from the Middle Neolithic in Germany. Archaeol Anthropol Sci. 2013;6: 199–204.
46. R Core Team (2012) R.: A language and environment for statistical computing. R Foundation for Statistical Computing. Vienna, Austria. Available:http://www.R-project.org/.
47. Ward JH. Hierarchical Grouping to Optimize an Objective Function. Journal of the American Statistical Association 1963;58: 236–244.
48. Knipper C, Maurer A-F, Peters D, Meyer C, Brauns M, Galer SJG, et al. Mobility in Thuringia or mobile Thuringians: a strontium isotope study from Early Medieval Central Germany. In: Kaiser E, Burger J, Schier W, editors. Population dynamics in prehistory and early history. New approaches using stable isotopes and genetic. Berlin, Boston: de Gruyter; 2012. pp. 287‒310.
49. Müller-Sohnius D. 87Sr/86Sr for isotope standards of Eimer and Amend (E&A), modern seawater strontium (MSS), and the Standard Reference Material (SRM) 987: development of interlaboratory mean values, procedures of adjusting, and the comparability of results Geologica Bavarica 110, 2007. pp. 1‒56.
50. Longin R. New method of collagen extraction for radiocarbon dating. Nature. 1971;230: 241–242.
51. Brown TA, Nelson DE, Vogel JS, Southon JR. Improved collagen extraction by modified Longin method. Radiocarbon. 1988;30: 171‒177.
52. Oelze VM, Siebert A, Nicklisch N, Meller H, Dresely V, Alt KW. Early Neolithic diet and animal husbandry: stable isotope evidence from three Linearbandkeramik (LBK) sites in Central Germany. J Archaeol Sci. 2011;38: 270‒279.
53. Paul D, Skrzypek G, Fórizs I. Normalization of measured stable isotopic compositions to isotope reference scales ‒ a review. Rapid Commun Mass Spectrom. 2007;21: 3006–3014.
